# Emerging strategies to improve heat stress tolerance in crops

**DOI:** 10.1007/s42994-024-00195-z

**Published:** 2025-01-24

**Authors:** Jiawei Xiong, Hao Wang, Zhaohui Zhong, Shigui Li, Peng Qin

**Affiliations:** State Key Laboratory of Crop Gene Exploration and Utilization in Southwest China, Rice Research Institute, Sichuan Agricultural University, Chengdu, 611130 China

**Keywords:** Heat stress, High temperature, Crops, Thermotolerance, Thermotolerance breeding

## Abstract

The heat stress (HS) response in plants involves complex processes at the molecular, cellular, and whole-organism levels. Sensitivity to HS differs based on the species and developmental stage of the plant, making it challenging to define HS and its impacts. Efforts to enhance HS tolerance by traditional breeding are constrained by limited genetic resources, but understanding the mechanisms that regulate HS responses can enable efforts to improve heat tolerance by precision breeding and gene editing. Here, we review recent research on the effects of HS on major cereal crops at different developmental stages and identify key genes potentially involved in the HS response, offering insight for precision molecular breeding. Additionally, we discuss the use of favorable natural variants and gene editing to improve crop tolerance to HS, emphasizing the value of alleles involved in thermomemory, combined stress tolerance, and the stress response–growth balance. This review aims to summarize recent advancements in understanding HS responses in crops, highlighting potential avenues for generating heat-tolerant crops.

**Supplementary Information:**

The online version contains supplementary material available at 10.1007/s42994-024-00195-z.

## Introduction

**Fig. 1:**
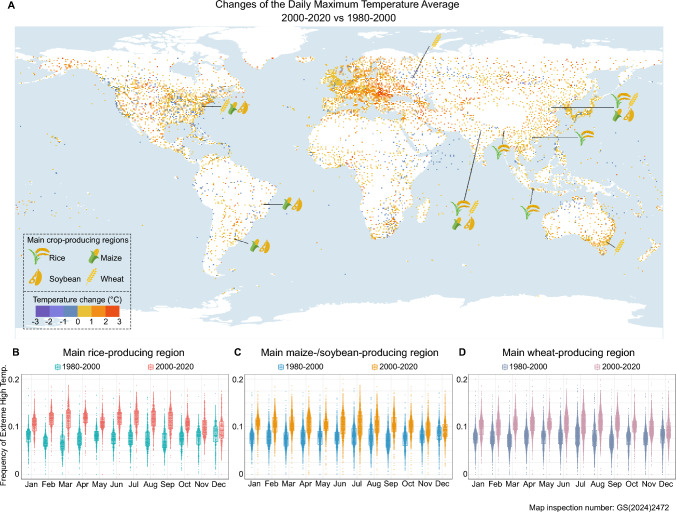
Trends in global temperature change. **A** Changes in the daily maximum temperature average (°C) during 2000–2020 relative to 1980–2000. The temperature data were obtained from NCEI (https://www.ncei.noaa.gov/), NEO (https://neo.gsfc.nasa.gov/), GES DISC (https://disc.gsfc.nasa.gov/), and ECMWF (https://cds.climate.copernicus.eu/). Only the data from stations located between 66°34′ N and 66°34′ S latitude and at an altitude below 1 km were included in the calculations. In the figure, the main production regions of major food crops (rice, maize, wheat, soybean) are marked based on the top five countries in production quantity for each crop, as published by the FAO in 2022 (https://www.fao.org/faostat/en/#data/QCL). The main producing countries are as follows: for rice, China, India, Bangladesh, Indonesia, and Vietnam; for maize and soybean, the USA, China, Brazil, Argentina, and India; and for wheat, China, India, Russia, the USA, and Australia. **B** Frequency of extreme high temperatures in the main rice-producing region during the periods 1980–2000 and 2000–2020. A hot day is defined as a day when the daily maximum temperature is higher than its historical 90th percentile for that specific calendar day. These daily-based 90th percentiles are determined by ranking historical (1980–2020) 15 day samples surrounding each day (7 days before and after, i.e., a total of 15 × 40 = 600 days) (Wang et al. 2020a). Each point in the figure represents the ratio of extreme high-temperature days to the total number of observed days for a specific time period (e.g., all January days from 1980–2000) at a given station, indicating the frequency of hot extremes. **C** Frequency of extreme high temperatures in the main maize-/soybean- producing region during the periods 1980–2000 and 2000–2020. **D** Frequency of extreme high temperatures in the main wheat-producing region during the periods 1980–2000 and 2000–2020. All monthly frequencies of extreme high temperatures in 2000–2020 are significantly higher than those in 1980–2000 (*P* < 0.0001, figure not shown), as determined by the Wilcoxon rank-sum test

Temperature is a primary environmental factor influencing plant growth, development, and yield (Janni et al. 2020). With the growth of the population and industrial development, global warming has become an issue that cannot be ignored (Quint et al. 2016). According to a report by the National Aeronautics and Space Administration (NASA, https://climate.nasa.gov/vital-signs/global-temperature/), the Earth’s average surface temperature has risen by approximately 0.9 °C since the late nineteenth century. The average maximum daily temperature in 2000–2020 was significantly higher than it was in 1980–2000, indicating that our world continues to get hotter (Fig. 1A). By the year 2100, temperatures are projected to increase by approximately 1.0–5.7 °C (IPCC 2023a; IPCC 2023b). With the continued increase in global temperatures (Fig. 1A), high temperatures pose a serious threat to global crop production (Heino et al. 2023; Janni et al. 2020). Since 1980, global maize and wheat yields have decreased by approximately 4–5% compared to scenarios without climate change (Lobell et al. 2011). With every 1 °C increase in average temperature, global yields of wheat, rice, maize, and soybean decrease by approximately 6.0%, 3.2%, 7.4%, and 3.1%, respectively (Zhao et al. 2017). Meanwhile, with the world’s population expected to reach 9 billion by mid-century, a 70% increase in food production by 2050 is estimated to be necessary to meet the growing demand (Bita and Gerats 2013; Godfray et al. 2010). Hence, it is urgent to explore heat-resistance genetic resources, unravel the molecular mechanisms underlying heat resistance, and breed heat-tolerant crop varieties.

Research into plant responses to high temperatures has generally focused on the model plant *Arabidopsis thaliana*, although recent progress in crops provides a basis for breeding heat-resistant varieties (Ding and Yang 2022; Kan et al. 2023; Li et al. 2023b; Xu et al. 2021b). However, available genetic resources for breeding remain limited (Fig. 1; Table 1; Table 2; and Supplemental Table S1), necessitating deeper exploration of favorable natural alleles. This article reviews recent research on how HS affects major cereal crops like rice (*Oryza sativa*), maize (*Zea mays*), and wheat (*Triticum aestivum*) at different developmental stages and highlights genes that may play a role at each stage (Supplemental Table S1), aiming to provide potential targets for further research. We summarize current research on crop HS and explore ways to improve crop heat tolerance through efficient screening of natural variations and genome editing.

**Table 1:** Number of genes regulating crop heat response

Crops	Genes	Developmental stages		
		Vegetative	Reproductive	Grain filling
Rice	77	53	28	12
Maize	14	11	4	0
Wheat	22	18	4	4

**Table 2:** Reported thermotolerance alleles in crops

Genes/Proteins	Functions	Thermotolerance alleles	References
*TT1* (*LOC_Os03g26970*); The α2 subunit of the 26S proteasome	Eliminating cytotoxic denatured proteins	*TT1*^*CG14*^, from *O. glaberrima*	Li et al. (2015)
*TTL1* (*LOC_Os01g66970*); A C3HC4 type transcription factor	Loss-of-function mutations in *TTL1* enhances heat tolerance, and causes an increase in grain size	*TTL1*^*hapL*^, mainly distributed in the *indica* subpopulation of *O. sativa*	Lin et al. (2023b)
*TT2* (*Os03g0407400*); A heterotrimeric G-protein γ subunit	Inhibiting wax synthesis under HS through SCT1	*TT2*^*HPS32*^, from the *japonica* subpopulation of *O. sativa*	Kan et al. (2022)
*OsHTG3* (*LOC_Os03g06630*); A heat shock transcription factor	Regulating *OsJAZ9, OsJAZ12* and other heat response genes to increase plant heat resistance	*HTG3*^*M−*^, mainly distributed in the *indica* subpopulation of *O. sativa*	Cheng et al. (2015), Wu et al. (2022)
*SLG1* (*LOC_Os12g39840*); The cytosolic tRNA 2-thiolation protein 2	SLG1-mediated thermotolerance is positively correlated with thiolated tRNA levels	*SLG1*^*Ind*^, mainly distributed in the *indica* subpopulation of *O. sativa*	Xu et al. (2020c)
*OsGSA1* (*LOC_Os03g55040*); A UDP-glucosyltransferase	Facilitating the accumulation of flavonoid glycosides and anthocyanins	*GSA1*^*WYJ*^*,* from the *japonica* subpopulation of *O. sativa*	Dong et al. (2020)
*SRL10* (*LOC_Os10g33970*); A double-stranded RNA-binding protein	Interacting with catalase isozyme B (CATB) and augmenting its H_2_O_2_ scavenging capacity	*SRL10*^*hap3*^, mainly distributed in the *indica* and *Aus* subpopulations of *O. sativa*	Wang et al. (2023b)
*HTH5* (*LOC_Os05g05740*); A pyridoxal phosphate homeostasis protein	Reducing ROS accumulation by increasing the pyridoxal 5’-phosphate (PLP) content	*HTH5*^*HTT3*^, from *O. rufipogon*	Cao et al. (2022)
*SUS3* (*LOC_Os07g42490*); A sucrose synthase	The upregulation of *SUS3* during ripening enhances heat tolerance in rice	*SUS3*^*haba*^, from the *japonica* subpopulation of *O. sativa*	Takehara et al. (2018)
*OsNRT2.3* (*LOC_Os01g50820*); A high-affinity nitrate transporter	Maintaining high yield and high nitrogen use efficiency under high temperatures	*OsNRT2.3*^*HTNE−2*^, mainly distributed in the *Aus* subpopulation of *O. sativa*	Zhang et al. (2022b)
*TT3.1* (*LOC_Os03g49900*); A RING finger ubiquitin E3 ligase *TT3.2* (*LOC_Os03 g49940*); A chloroplast precursor protein	TT3.1 translocates during HS, leading to the ubiquitination of TT3.2 for vacuolar degradation. This process protects thylakoids from HS	*TT3*^*CG14*^, from *O. glaberrima*	Zhang et al. (2022a)
*TaSG-D1* (*TraesCS3A02G136500*); A STKc_GSK3 kinase	Enhancing phosphorylation and stability of downstream target TaPIF4	*TaSG-D1*^*E286K*^, from *Triticum sphaerococcum*	Cao et al. (2024)

## The effects of HS

Previous studies have predominantly focused on the frequency and effects of summer heatwaves (Kan et al. 2023; Wang et al. 2020a; Xu et al. 2021b). However, a focus on summer heat may overlook broader effects on crop development because crops growth periods differ in different crops and in the same crop in different planting regions. For example, in rice single- and double-cropping systems, regional planting conditions significantly affect the growth period. Moreover, heat sensitivity varies among crops and across different developmental stages in the same crop. In rice, HS significantly affects germination, root development, tillering, reproductive growth, and grain filling. However, in agricultural production, the effect of HS is most pronounced during critical stages such as tillering, reproductive growth, and grain filling, significantly affecting crop yield and quality. Research on beneficial alleles regulating heat tolerance at these stages is currently limited. Given these complexities, there is a critical need to thoroughly understand crop heat tolerance mechanisms across development to facilitate the development of heat-resistant varieties.

To explore the effect of HS on different crops and planting regions, we analyzed the frequency of extreme high-temperature events in major cereal crop production regions for rice, maize, and wheat (https://www.fao.org/faostat/en/#data/QCL). (Fig. 1). This analysis showed a substantial rise in extreme high-temperature events across different months in all cereal-producing regions from 2000–2020 compared to 1980–2000 (Fig. 1 B-D). The increasing frequency of extreme heat events during various developmental stages across all crops underscores the importance of investigating relative heat stress (Heat stress is defined relative to the developmental stage, as different stages exhibit varying sensitivities to high temperatures). A more comprehensive understanding of the hazards of extreme heat will enable more targeted research and cultivar improvement. In the next sections, we explore the effects of HS at different critical stages for grain production.

### Effects of HS at the tillering stage

Tillering is a determinant of rice yield as the number of tillers correlates with the number of panicles, making it an useful indicator for breeding varieties that maintain yield in the face of heat stress (Prasanth et al. 2017). Exposure to high temperatures usually decreases tiller and panicle numbers (Oh-e et al. 2007), and this effect is more severe in *japonica* rice compared to *indica* rice (Wang et al. 2016).

The essential macronutrients nitrogen (N), phosphorus (P), and potassium (K) have notable effects on tillering (Wang et al. 2020b). For example, N has a affects axillary bud growth, thereby affecting rice tillering and grain yield (Wang et al. 2020b). Under high night temperature conditions, rice varieties containing the HIGH TEMPERATURE RESISTANT AND NITROGEN EFFICIENT-2 (HTNE-2) allele of the nitrate transporter gene *NRT2.3* show heat tolerance due to more translation of *OsNRT2.3b* mRNA (Zhang et al. 2022b). Additionally, previous studies have found that heat treatment suppresses the expression of several nitrate transporter genes, including *NRT2.2*, *NAR2.1*, *NRT2.1*, and *NRT2.3* (Feng et al. 2011). However, the molecular mechanisms underlying the effect of high temperature on N uptake, as well as whether and how high temperature affects crop absorption of other elements, remain to be studied.

### Effects of HS at the reproductive stage

Rice plants are more susceptible to HS at the reproductive stage—which encompasses panicle initiation, male and female gametophyte development, anthesis, pollination, and fertilization—than at the vegetative stage (Arshad et al. 2017; Xu et al. 2021b).

Plants exposed to high temperatures produce fewer spikelets, and the effects are most severe when temperatures remain high throughout the day and night (Xu et al. 2020b, 2021b). Ensuring spikelet development under high-temperature conditions is critical for achieving optimal yield. A transposable element-derived transcription factor, THERMOSENSITIVE BARREN PANICLE (TAP), has been shown to positively regulate panicle and spikelet development under high temperatures (Zhang et al. 2023). The expression and protein abundance of TAP in rice are increased under elevated temperatures, and TAP directly promotes the expression of *OsYABBY3* (*OsYAB3*), *OsYAB4*, and *OsYAB5*, which encode key transcriptional regulators in panicle and spikelet development (Cai et al. 2023; Zhang et al. 2023). The MADS-box protein OsMADS8 regulates pistil number and ovule initiation by modulating the expression of downstream genes in flower development pathways, such as *OsYABBY* homologs, in a temperature-dependent manner (Shen et al. 2023). Mitochondria, as the energy hub of the cell, play a crucial role in spikelet development under HS. In maize, the mitochondrion-localized ATP-dependent metalloprotease FILAMENTATION TEMPERATURE-SENSITIVE H 10 (ZmFTSH10) sustains maize organogenesis in HS by maintaining reproductive meristem redox status and auxin homeostasis (Liu et al. 2019b). Similaly, EXTRA GLUME1 (EG1), a PLA1-type phospholipase, promotes robustness of flowering during temperature fluctuations by maintaining the expression of floral identity genes through a high-temperature-dependent mitochondrial lipid pathway (Zhang et al. 2016).

Spikelet fertility affects the number of filled grains at maturity, thus making a substantial contribution to the final grain yield (Li et al. 1998). During the meiotic division stage in pollen mother cells (PMCs), high temperatures can lead to premature degradation and disintegration of tapetal cells, affecting the nutrient supply to microspores and the formation of pollen walls, resulting in pollen grain abortion (Feng et al. 2018; Oshino et al. 2007; Xu et al. 2021b). Developing anthers are significant resource sinks, and heat stress adversely affects the development of tapetum cells and microspores, impacting the synthesis of DNA, carbohydrates, proteins, and lipids (Santiago and Sharkey 2019; Sita et al. 2017). In maize, HSP101, a member of the HSP100 family, is induced by heat stress and participates in the loading of RADIATION SENSITIVE 51 (ZmRAD51), a recombinase essential for double-strand break (DSB) repair during meiosis. Consistent with these roles, overexpression of HSP101 in anthers leads to robust microspores with enhanced heat tolerance (Li et al. 2022c)*.* Additionally, INVERTASE ALKALINE NEUTRAL 6 (INVAN6), a maize cytosolic invertase predominantly found in PMCs, specifically hydrolyzes sucrose and regulates glucose metabolism and transport genes to maintain anther glucose homeostasis, alleviating HS and supporting normal meiosis in PMCs (Huang et al. 2022).

HS also inhibits anther dehiscence, leading to less available pollen on the stigma (Matsui et al. 1997; Prasad et al. 2006). HS disrupts anther water metabolism, reducing the ability of pollen to absorb water and expand (Matsui et al. 2005, 2000; Matsui and Omasa 2002; Wilson et al. 2011). In addition, HS increases the ROS levels in the plant, leading to hardened anther walls and abnormal septal rupture, further hindering anther dehiscence (Jagadish et al. 2010; Kobayashi et al. 2011).

Pollen tube elongation into the ovary during rice pollination is crucial for fertilization and directly impacts yield. HS reduces fertilization rates by disrupting the cellular calcium ion balance, which is essential for the directional elongation, reception, and rupture of pollen tubes (Gao et al. 2022; Xu et al. 2017). During pollen tube reception by the pistil, the pollen tube induces an increase in calcium ion (Ca^2+^) concentration within the embryo sac’s synergids. The elevated Ca^2+^ guides the pollen tube to penetrate the synergids and rupture to release sperm. HS can prematurely trigger fluctuations in Ca^2+^ concentration, leading to the loss of guidance for pollen tube directional elongation (Santiago and Sharkey 2019). The ROS accumulation induced by HS in plant cells can also inhibit pollen tube elongation (Muhlemann et al. 2018; Zhang et al. 2018a).

Overall, high-temperature-induced sterility is closely associated with ROS bursts, and some proteins are known to support fruiting under heat stress by maintaining ROS balance during reproductive development. For example, HEAT TOLERANCE AT THE HEADING STAGE ON CHROMOSOME 5 (HTH5), a pyridoxal phosphate homeostasis protein, lowers ROS accumulation by increasing heat-induced pyridoxal 5′-phosphate (PLP) content, thereby improving the seed-setting rate for rice plants under high temperature during the heading stage (Cao et al. 2022). ENHANCED DISEASE SUSCEPTIBILITY 1 (EDS1) enhances thermotolerance by interacting with and promoting catalase-mediated H_2_O_2_ scavenging, thus controlling ROS homeostasis (Liao et al. 2023). HSP60-3B protects pollen development by regulating starch accumulation and ROS levels under high temperatures (Lin et al. 2023a). In addition, overexpression of *OsNCED1* improves the thermotolerance of rice at the heading and flowering stages by enhancing antioxidant capacity (Zhou et al. 2022a).

### Effects of HS at the grain-filling stage

High temperatures during the grain-filling stage significantly reduce grain weight and quality. Plants under HS avoid or mitigate stress through phenotypic plasticity, often leading to a shortened grain-filling stage (Waqas et al. 2021). Consequently, HS shortens the time for crops to capture resources during grain filling, ultimately reducing grain yield and quality (Li et al. 2022a; Shi et al. 2017; Sita et al. 2017). High temperatures during grain filling affect the content and composition of grain starch (Li et al. 2017a; Sita et al. 2017; Wang et al. 2015; Xu et al. 2021b). Cereals regulate starch synthesis through a complex network involving enzymes such as sucrose synthase and soluble starch synthase. The *Wx* (*waxy*) gene, a key regulator of amylose biosynthesis, maintains stable amylose levels and enhances grain quality under HS when its pre-mRNA splicing efficiency is enhanced (Zhang et al. 2014). While constitutive suppression of *OsMADS7* stabilizes amylose levels during HS, it reduces spikelet fertility. Targeted suppression of *OsMADS7* specifically in the endosperm mitigates this drawback, balancing quality and fertility (Zhang et al. 2018b). The NAC transcription factors ONAC127 and ONAC129 regulate grain filling by influencing sugar transport under HS conditions. Knockout and overexpression plants exhibit incomplete grain filling and grain shrinkage, with more severe effects observed under HS (Ren et al. 2021). Polycomb repressive complex 2 (PRC2) plays a critical role in endosperm development in cereals. Notably, OsFIE1, a core PRC2 component, regulates seed size under HS by modulating early endosperm development (Dhatt et al. 2021; Folsom et al. 2014).

In rice, grain chalkiness is a prominent symptom of HS during grain filling (Qin et al. 2021b; Xu et al. 2021b). Elevated temperatures enhance α-amylase expression and activity, leading to chalky grains due to excess α-amylase accumulation, even without direct HS exposure (Nakata et al. 2017). OsbZIP58 promotes the transcription of genes for seed storage proteins and starch synthesis enzymes while repressing α-amylase genes under HS. Loss of OsbZIP58 function results in abnormal grain morphology, higher chalkiness, and reduced starch and amylose levels (Xu et al. 2020a). Leaf-derived abscisic acid (ABA) also regulates seed development, with DEFECTIVE GRAIN-FILLING1 (DG1), a rice MATE transporter, facilitating long-distance ABA transport to activate starch synthesis genes essential for grain filling. DG1-mediated ABA transport efficiency and grain-filling phenotypes are temperature-sensitive, impacting grain filling and chalkiness under HS (Qin et al. 2021b). Additionally, OsCG5, a grain-specific protein with an undefined function, shows a negative correlation between its transcript levels and grain chalkiness under HS, suggesting a potential regulatory role in grain quality (Chandran et al. 2022).

### Effects of HS on the chloroplasts

Chloroplasts supply the energy needed for crop growth and reproductive development and are highly susceptible to heat stress. The plastid-encoded RNA polymerase (PEP) plays a crucial role in chloroplast transcription. WHITE LEAF AND PANICLE 2 (WLP2), a plastid-encoded RNA polymerase-associated protein, protects chloroplast development from HS (Lv et al. 2017). Photosynthesis itself is sensitive to heat stress. Transgenic rice plants overexpressing *RUBISCO ACTIVASE* (*RCA*) exhibit greater thermotolerance than wild-type plants (Wang et al. 2010). Overexpressing both *Rubisco* and *RCA* enhances photosynthesis, and ultimately yields, in rice under heat stress conditions (Qu et al. 2021). The photosystem II (PSII) complex subunit D1 is particularly vulnerable to heat stress, and expressing it from genes driven by artificial promoters can increase thermotolerance and grain yield in rice (Chen et al. 2020). In wheat, the nucleus-encoded small heat-shock protein sHSP26 protects PSII under heat stress conditions, thereby enhancing heat resistance (Chauhan et al. 2012). HIGHER YIELD RICE (HYR), an AP2/ERF transcription factor, is a master regulator that activates photosynthesis-related genes, enhancing both photosynthesis and yield in rice under normal and high-temperature conditions (Ambavaram et al. 2014). INTERMEDIATE FILAMENT (IF) confers heat tolerance by stabilizing the photosynthetic machinery under heat stress (Soda et al. 2018). Ta2CP, a 2-cysteine peroxiredoxin in wheat, participates in chlorophyll metabolism by interacting with PROTOCHLOROPHYLLIDE REDUCTASE B (TaPORB) (Mishra et al. 2021).

Chloroplasts are also implicated in cellular signal transduction and responses to heat stress, although the precise mechanisms remain unclear. The recent discovery of the TT3.1–TT3.2 module elucidated a link for heat signals between the plasma membrane and chloroplasts in rice, unveiling a mechanism of response to extreme heat (Zhang et al. 2022a). TT3.1 is a transmembrane E3 ligase that acts as a thermosensor, moving from the plasma membrane to the endosomes under heat stress. There, TT3.1 recruits and ubiquitinates the chloroplast precursor protein TT3.2, which is then degraded in the vacuole, reducing TT3.2 accumulation-induced chloroplast damage and enhancing heat tolerance in rice (Zhang et al. 2022a). Since TT3.1 and TT3.2 are conserved across many crops, they offer potential for improving heat tolerance and addressing food security issues due to global warming.

## Approaches to resolve HS challenges

**Fig. 2:**
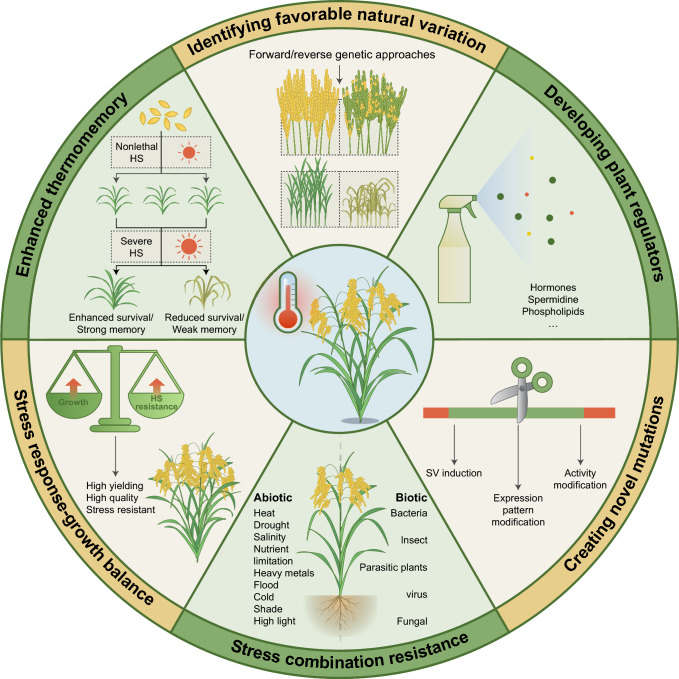
Approaches to address heat stress (HS) challenges. Diagram of potential approaches to mitigate the harmful effects of HS on crops. Identifying favorable alleles from germplasm resources through forward or reverse genetics and breeding heat-tolerant varieties is fundamental to overcoming the effects of HS. Given the variable environment, exploring rare alleles with enhanced functions, such as thermomemory, combined stress tolerance, and stress response–growth balance, is essential. Genome editing offers a rapid, targeted method to create beneficial mutations. Additionally, developing low-cost, eco-friendly plant regulators provides a complementary approach to alleviate the effects of HS, especially as understanding HS mechanisms and mass-producing heat-tolerant crops remain long-term challenges

Cultivation practices can partially mitigate the effects of HS on crop yield and quality, but the increasing unpredictability of high-temperature events poses a growing challenge (Fig. 1). In-depth research on crop HS-response mechanisms is needed for gene identification and natural variation mining, as the number of thermotolerance alleles currently available for breeding is very limited (Table 2). Most known heat tolerance–related natural variations in rice are in the *indica* subpopulation, but a total of only 11 alleles has been identified (Table 2). As increases of daily maximum temperatures are more pronounced in *indica* rice–producing regions (Fig. 1A), it is critical to identify more robust heat-tolerance alleles for *indica* cultivars. Here, we discuss methods to rapidly identify favorable natural variants. Given the current complex and variable natural environment, rare alleles with enhanced functions, such as in thermomemory, combined stress tolerance, and stress response–growth balance, would be especially valuable. Finally, we discuss the potential of using gene editing to obtain stronger alleles, providing new strategies for crop improvement (Fig. 2).

### Identifying favorable natural variants

Currently, the most effective approach to address HS is the development of thermotolerant crop varieties. Given the limited progress in agronomic management and conventional breeding for thermotolerance (Driedonks et al. 2016), identifying heat-resistance genes or quantitative trait loci (QTL) and incorporating them into breeding programs has become an urgent priority.

In rice, numerous QTL conferring thermotolerance at various developmental stages, such as seedling, booting, flowering, and grain filling, have been identified and validated (Xu et al. 2021b). At the seedling stage, notable QTL include *OsHTAS*, a dominant major locus on chromosome 9 conferring tolerance to 48 °C in rice seedlings (Liu et al. 2015; Wei et al. 2013), and *RLHT5.1*, which influences root length under HS (Kilasi et al. 2018). *qPF4* and *qPF6*, identified by Xiao et al., enhance pollen fertility, while *SSR4* and *SSR10* contribute to spikelet fertility under heat stress (Xiao et al. 2011). Zhao et al. identified 11 QTL using chromosome segment substitution lines, including three novel loci (*qPSL*^*ht*^*4.1, qPSL*^*ht*^*7, qPSL*^*ht*^*10.2*) linked to spikelet fertility and flowering time (Zhao et al. 2016). Ye et al. pinpointed two major QTL, *qHTSF1.1* and *qHTSF4.1*, in F2 populations derived from IR64 and N22 (Ye et al. 2012). Using QTL-seq, Nubankoh et al. detected three loci (*qSF1, qSF2, qSF3)* influencing spikelet fertility (Nubankoh et al. 2020). Ps et al. identified five QTL, including a novel locus (*qSTIPSS9.1*), associated with spikelet fertility and yield (Ps et al. 2017). Cheng et al. reported four loci (*qSF4, qSF5, qSF6, qSF11*) with significant contributions to thermotolerance, particularly *qSF4* and *qSF6*, which reduce phenotypic disparities between normal and stress conditions (Cheng et al. 2012). Several QTL related to thermotolerance during the grain-filling stage have also been identified. For example, *Appearance quality of brown rice 1* (*Apq1*) has been mapped to a 19.4-kb region, with the underlying gene, *sucrose synthase 3* (*Sus3*), having been cloned (Takehara et al. 2018). Wada et al. identified 10 QTL for white-back and basal-white grains caused by heat stress, with qWB8 improving grain quality under elevated temperatures (Wada et al. 2015). Additionally, the effects of *qWB6*, *qWB9*, and *qMW4.1* have been validated in multiple environments (Kobayashi et al. 2013; Miyahara et al. 2017)*.*

Despite these advancements, the identification of causal genes underlying these QTL remains challenging, with only a few successfully cloned and functionally validated to date (Table 2). In crop research, there are two primary methods for studying phenotype and genetic variation: linkage analysis and genome-wide association studies (GWAS) (Bettgenhaeuser and Krattinger 2019). These forward-genetics methods aim to identify molecular markers linked to traits of interest, and both detect major-effect quantitative trait loci (QTLs) well. However, both methods are challenged by population characteristics, making direct gene localization difficult. Validation and fine mapping of QTLs require the time-consuming and labor-intensive construction of secondary mapping populations. For instance, cloning the maize southern leaf blight resistance genes *Ht2/Ht3* took nearly 10 years (Yang et al. 2021), and cloning the rice bacterial blight resistance gene *Xa21* required a decade (Bettgenhaeuser and Krattinger 2019). There were ~ 20 years between the discovery of the rice bacterial blight resistance gene *Xa7* and its cloning (Chen et al. 2021b). The exploration of heat-tolerance genes from a forward-genetic perspective has always been a challenging process, involving complex genetic backgrounds, diverse phenotypes, and their interactions with environmental conditions (Zhang et al. 2022a). Therefore, some researchers opt to utilize reverse genetics instead, starting from mutations in a candidate gene and looking for related phenotypic changes.

Genomic structural variations (SVs) are widely present in the genome, ranging in size from 50 bp to over megabases (Alkan et al. 2011). SVs can influence agronomic traits by altering gene coding sequence, gene copy number, or the composition or position of cis-regulatory sequences, thereby affecting gene expression (Qin et al. 2021a; Weischenfeldt et al. 2013). Compared to single nucleotide polymorphisms (SNPs) and insertions/deletions (InDels), SVs have a greater impact on the genome sequence. Individual genomic differences caused by SVs are 3–10 times higher than those caused by single nucleotide variants (SNVs) (Xie et al. 2023). Thus, SVs are attractive analytical targets for studying complex traits, especially when involving key genes or critical regulatory regions, where the phenotypic effects of SVs may be more pronounced. Identifying SVs from genetically diverse rice materials and exploring the correlation between SVs and gene function and phenotype from a reverse-genetic perspective, combined with transcriptomic data before and after heat treatment, for instance, theoretically enables rapid exploration of favorable natural variations regulating heat tolerance phenotypes in rice.

Any resulting candidate favorable alleles can be introduced into recipient cultivars using DNA markers linked to the QTL. For instance, near-isogenic lines (NILs) introgressed with thermotolerance alleles such as *TT1*^*CG14*^*, TT2*^*HPS32*^*, TT3*^*CG14*^, and *SLG1*^*Ind*^ exhibit improved grain quality under HS compared to conventional recipient cultivars (Kan et al. 2022; Li et al. 2015; Xu et al. 2020c; Zhang et al. 2022a). Traditional breeding methods are often time-consuming and inefficient, but transgenic introduction of beneficial mutations offers a straightforward and effective alternative in various crops. For instance, overexpression of genes like *OsIF*, *OsANN1*, *ZmCDPK7*, and *TaSG-D1* has conferred enhanced heat tolerance in rice, maize and wheat (Cao et al. 2024; Qiao et al. 2015; Soda et al. 2018; Zhao et al. 2021). However, public health and environmental concerns surrounding GM crops continue to hinder their commercialization and broader adoption (Zhang et al. 2018c). The advent of genome editing technology provides a promising approach for plant molecular breeding, allowing for the rapid introduction of natural thermotolerance variations through the development of plants with targeted mutations (Gao 2019). In addition, genome editing has helped identify the molecular mechanism of heat resistance; the roles of *ONAC023, ZmHUG1*, and *TaHSFA1* in crops thermotolerance were validated using the CRISPR-Cas9 system (Chang et al. 2024; Wang et al. 2023a; Xie et al. 2022). In later sections, we will focus on the significant potential of genome editing technology in creating novel variations. Another major challenge in developing thermotolerant crop varieties lies in evaluating the breeding value of thermotolerant alleles. To determine the impact of genetic variations on crop thermotolerance and productivity, multi-year, multi-location field trials should be conducted, using elite cultivars within their corresponding ecological regions. These trials must comprehensively assess thermotolerance alongside fundamental agronomic traits, ensuring that improvements in thermotolerance are achieved with minimal or no compromise to other critical agronomic characteristics.

### Thermomemory in breeding

After brief exposure to stresses such as drought, salt, high light intensity, or heat, plants can acquire stress memory, which helps them respond strongly and rapidly when encountering the stress again (Crisp et al. 2016). For example, after HS priming, i.e., an initial exposure to HS, plants acquire an improved response to a later HS (Charng et al. 2007, 2006). This thermomemory improves survival in cases where recurring HS would normally be lethal to the plant (Charng et al. 2007, 2006; Stief et al. 2014). HS memory and recovery are controlled at the transcriptional, epigenetic, post-transcriptional, and metabolic levels (Balazadeh 2022; Crisp et al. 2016; Kumar and Wigge 2010; Ling et al. 2018; Liu et al. 2019a; Sedaghatmehr et al. 2016; Serrano et al. 2019; Thirumalaikumar et al. 2021; Zhong et al. 2013).

Transcriptional memory arises from the coordinated interplay between transcription factors (TFs) and chromatin regulatory factors, which affect histone methylation and nucleosome remodeling. Studies in *Arabidopsis* have implicated HSFA2 and HSFA3 as key transcription factors in thermomemory. The transient binding of HSFA2 to the promoters of thermomemory genes induces their expression and facilitates the long-term enrichment of histone methylation marks at these loci (Lämke et al. 2016). Additionally, HSFA2 can heterodimerize with HSFA3 to induce rapid expression of heat-responsive genes via H3K4me2 or H3K4me3 (Friedrich et al. 2021). However, the mechanism by which HSFA2 and HSFA3 recruit chromatin-modifying factors to target loci remains to be elucidated.

The establishment of thermomemory in *Arabidopsis* also involves post-transcriptional mechanisms, such as splicing memory formation and post-transcriptional gene silencing. In response to high temperatures, selective splicing of heat-responsive genes is inhibited in *Arabidopsis* (Shalgi et al. 2014). In addition, *Arabidopsis* plants primed with heat initiation were reported to regain effective splicing upon subsequent exposure to high temperatures, whereas unprimed plants tended to accumulate transcripts retaining introns under similar high-temperature conditions (Ling et al. 2018). miRNA-dependent gene silencing also contributes to thermomemory. Heat-induced activation of miR156 sustains the expression of thermomemory genes by inhibiting the squamosa promoter binding-like TFs SPL2, and SPL11 (Stief et al. 2014).

Heat shock proteins are regulated by heat shock transcription factors and participate in the formation of thermomemory; heat shock protein stability is also crucial for the continuation of thermomemory. In *Arabidopsis*, the FKBP family member ROF1 forms a ROF1–HSP90.1–HSFA2 complex that regulates the expression of *sHSPs* (Meiri and Breiman 2009; Schramm et al. 2006; Sedaghatmehr et al. 2019). Although ROF1 itself is not directly related to the formation of thermomemory, its increased protein levels during the memory phase are crucial for extending thermomemory (Thirumalaikumar et al. 2021). HSP101 enhances the translation of *HSA32* during the recovery phase after heat priming, and HSA32 in turn inhibits the degradation of HSP101 protein. Together, they form a positive feedback loop to promote thermomemory (Charng et al. 2006; Wu et al. 2013). Studies in rice have found similar HSP101–HSA32 thermomemory regulation resulting in the maintenance of high levels of these proteins during the recovery phase after heat shock, thus enhancing thermomemory capacity in the *japonica* rice cultivar Nip (Lin et al. 2014).

Some studies indicate that memory can persist across generations, a process known as adaptive transgenerational plasticity (Crisp et al. 2016; Herman and Sultan 2011; Liu et al. 2019a; Niu et al. 2024). One strategy to enhance heat tolerance in crops may be to bolster thermomemory, as plants with robust memory capabilities may respond more effectively to subsequent, potentially harsher heat exposures after moderate heat exposure and could transmit this ability to the next generation. However, there are still many constraints in understanding the mechanism of thermomemory and mining for favorable alleles. Conducting large-scale heat shock treatments and subsequent phenotype identification poses significant challenges, making it difficult to rapidly identify favorable alleles. However, applying inducers early during rice seed germination can subject the seeds to mild stress, leading to more effective responses to recurring stress after seedling emergence (Ali et al. 2017). The phenotypes triggered by this type of seed treatment in rice is similar to stress memory phenotypes observed in *Arabidopsis*, suggesting that seed priming treatments could also be used to establish heat stress memory during seed germination (Schwachtje et al. 2019). Similar to heat-primed plants, the stress imprint generated by seed priming can be epigenetically marked on the genome (Srivastava et al. 2021). Large-scale treatment and phenotypic identification of seeds may facilitate the rapid discovery of beneficial alleles. Moreover, seed priming treatments are relatively easy to apply for agricultural production.

### Deciphering the mechanisms coordinating combined stress responses and growth

In nature, plants often encounter more than one environmental stress simultaneously, leading to a phenomenon termed ‘stress combination’. Compared to an individual stress, combined stresses pose a greater threat to plant growth and productivity (Mittler 2006; Suzuki et al. 2014). The diverse effects of various stress factors on plant physiology and metabolism can result in additive effects, leading to sharp declines in growth and productivity (Zandalinas and Mittler 2022). Indeed, when the number and complexity of stresses affecting plants increase, even if the level of each stress is relatively low, plant growth is significantly affected, sometimes leading to death (Sinha et al. 2024; Zandalinas et al. 2021). Some studies have also indicated positive interactions between two different stresses, such as the beneficial effect of moderate salt application in alleviating the damage caused by low-temperature stress (Zhou et al. 2022b). Plant responses to simultaneous exposure to multiple stress factors exhibit unique characteristics that cannot be simply extrapolated from the individual responses to each stress factor (Mittler 2006; Zandalinas et al. 2020, 2018). Therefore, we must assess the responses of plants to simultaneous stresses to accurately determine their response to the combined stress.

Crop plants in the field are often subjected to the combined stressors of high temperature and drought. HS leads to stomatal opening for cooling, but drought stress induces the opposite response to prevent water loss. Under combined drought and HS, leaf stomata remain closed, causing leaf temperatures to rise to dangerous or even lethal levels (Rizhsky et al. 2004). However, despite leaf stomatal closure under combined drought and HS, flower stomata remain open, enabling flowers to maintain transpiration and cool their reproductive tissues (Sinha et al. 2022). This suggests that even under two stresses with opposing physiological effects, there can be trade-off mechanisms. Moreover, genotypes tolerant to high temperature or drought as individual stressors may not necessarily exhibit tolerance to their combination (Cairns et al. 2013; Qaseem et al. 2019). For example, HS inhibits plant immunity, leading to more severe diseases in plants under high temperatures compared to normal temperatures (Hammoudi et al. 2018; Mang et al. 2012; Yang et al. 2023). Recent investigations suggest the existence of common pathways in plant responses to combined stressors, with significant overlaps in transcription factors and signaling pathways within the signal transduction mechanisms (Jing et al. 2024). Therefore, we propose that the best approach to addressing stress combinations in the future lies in finding the similarities that point to core components for stress-combination responses, identifying related superior alleles, unraveling mechanisms of the responses to combined stress, and finally breeding crops tolerant to the combined stressors.

As biological processes, plant growth and development often seem to be in opposition to plant stress responses. Plants must direct the use of their limited energy to maximize survival and reproduction, such that plants under stress reallocate energy and resources from growth to stress responses (Zhang et al. 2020). For crops, stress often leads to reduced yield. Therefore, high productivity and stress resistance may seem mutually exclusive. However, recent studies increasingly show that the balance between plant growth and defense is not solely determined by nutrient supply or resource limitations. For instance, overexpressing the GA biosynthesis gene GA5 along with DREB1A/CBF3 in *Arabidopsis* increased both biomass accumulation and survival under drought conditions (Kudo et al. 2019). The *jazQ phyB* mutant also exhibits strong growth and robust disease resistance simultaneously (Campos et al. 2016). These findings highlight the complexity of regulatory mechanisms and biological pathways that allow plants to dynamically adjust resource allocation to growth or stress resistance based on specific environmental challenges (Deng and He 2024; Zhang et al. 2020). The mechanisms may involve specific alterations in specific components. For example, cytokinin distribution patterns swiftly change under high salt stress, transitioning from aerial tissues to subterranean roots. Overexpression of the *AGO2* gene activates the cytokinin transport gene *BG3* via epigenetic modifications, mimicking the cytokinin distribution pattern observed under salt stress, thereby concurrently enhancing rice yield and salt resistance (Yin et al. 2020). IPA1 undergoes phosphorylation to facilitate the transition from growth and development to stress response, swiftly returning to a low-phosphorylation state that ensures normal rice development, thereby achieving both high productivity and stress resistance (Wang et al. 2018). Immunity and BR signals phosphorylate distinct sites of the common signal component BSU1, activating downstream signaling components GSK3 and MAPKs separately to initiate specific signal transduction pathways. This differential phosphorylation enables control of plant growth and immune processes through distinct signaling pathways. Importantly, mutating a specific phosphorylation site within one signaling pathway does not affect the functionality of the other signal pathway (Park et al. 2022).

Based on these results, we posit that advancing the production high-resistance, high-yield crops hinges on the "decoupling" of stress responses from growth and development. This underscores the critical importance of identifying pivotal components within stress and growth pathways, along with their tissue and time-specific interplay. Leveraging gene editing techniques like CRISPR/Cas9 should allow for precise modulation of specific regulatory elements, thereby facilitating the decoupling of stress response from growth and development.

### Creating novel mutations

To bring about revolutionary changes in crop improvement, it may be necessary to create advantageous mutations that do not exist in nature. Mutation breeding is widely used to generate new variations, but lacks precision. Due to challenges in screening for HS-related phenotypes, most of the mutants currently identified from mutant libraries are those with defects in heat responses (Chen et al. 2021a; Zhang et al. 2016, 2023). Therefore, there is an urgent need for more efficient and precise methods to generate favorable heat-tolerance variants.

Genome editing technology is a powerful tool to create specific mutations, and the CRISPR-Cas system is the most widely used approach. The CRISPR-Cas system consists of a single guide RNA (sgRNA) that is complementary to the target DNA and a nuclease to create a DNA double-strand break (DSB). The CRISPR-Cas9 (mainly SpCas9, which recognizes 5′-NGG-3′ PAM) and Cas12a (mainly LbCas12a, which recognizes 5′-TTTV-3′ PAM) are most commonly used in plants for genome editing (Jinek et al. 2012; Shan et al. 2013; Tang et al. 2017; Zetsche et al. 2015). Compared with LbCas12a, SpCas9 shows advantages in terms of platform extensibility. Based on the different SpCas9 nickases, the base editor (D10A) and prime editor (H840A) have been constructed (Anzalone et al. 2019; Gaudelli et al. 2017; Komor et al. 2016). By fusing the effector to dead SpCas9 (D10A, H840A), a gene regulation tool was developed to achieve gene repression or activation (Kiani et al. 2015). All of these tools should enable crop improvement to increase resistance to HS in three main ways: structural variation induction, gene expression level manipulation and enzyme activity level manipulation.

The CRISPR-Cas system can efficiently induce SVs, including genomic deletions (Li et al. 2022b; Song et al. 2022; Zhou et al. 2023), duplications (Lu et al. 2021) and inversions (Schwartz et al. 2020; Wang et al. 2022). By utilizing the DNA DSB repair pathway of non-homologous end joining (NHEJ) and homologous recombination (HR), a gene could be knocked-out or knocked-in to modulate the expression of heat response genes. Through NHEJ with InDels using SpCas9 or LbCas12a, single or multiplex gene knockout in plants has been achieved with high efficiency (Tang et al. 2019; Wang et al. 2017). Similarly, non-coding sequences including miRNA and circRNA loci could be modified through single sgRNA mutation or dual sgRNA deletion (Miao et al. 2019; Yang et al. 2019; Zhou et al. 2021). Recently, upstream open reading frames (uORFs) and micro-RNA encoded peptides (miPEPs) have been reported to have specific effects on downstream gene regulation, and thus influence phenotypes (Ormancey et al. 2024, 2021; Xing et al. 2020; Xue et al. 2023). Fine-tuning plant HS resistance through uORF or miPEPs by knockout or knockin could be a reasonable approach (Xue et al. 2023).

Gene expression levels can be manipulated by diverse CRISPR-Cas systems. Using the original CRISPR-Cas systems, the fine-tuning of gene expression can be achieved by promoter or 5’-UTR editing (Song et al. 2022; Zhou et al. 2023), with the new gene expression levels resulting in different phenotypes. For gene activation, knock-ins of strong promoters or enhancers can be used for gene promoters or 5’-UTR regions (Claeys et al. 2024; Vu et al. 2020). Editing in the 3’-UTR region can also cause gene upregulation, and different editing methods for UTR could result in gene expression changes.

The above methods for gene manipulation need full-function CRIPSR-Cas nucleases and cause DNA DSBs that may bring unwanted mutations and thus further influence traits. Combining a dead Cas nuclease with effector, gene regulation tools based on SpCas9 and LbCas12a have been developed to avoid DSBs of DNA (Li et al. 2017b; Tang et al. 2017). In addition, epigenomic editing is a newly developed technology. Combining a demethylation effector and methylation effector with dead Cas9, epigenomic tools could achieve cytosine demethylation and methylation, and thus further influence downstream gene expression (Hilton et al. 2015; Kleinstiver et al. 2019; Thakore et al. 2015). These gene regulation tools may provide avenues to increase the HS resistance.

To promote crop adaptation to HS, enzyme activity could be strengthened through mutation of key residues of HS-response genes by base editing and prime editing (PE). Base editing has been developed in CRISPR-Cas9, and -Cas12a systems, and it provides a basic tool to achieve efficient cytosine editing (like CBE and CGBE(Kurt et al. 2021)) and adenine editing (like ABE and AYBE) (Gaudelli et al. 2017; Komor et al. 2016; Kurt et al. 2021; Tong et al. 2023). Base editors induce nucleotide conversion without DSBs, which shows potential for direct evolution of thermotolerance-related gene. Combined with population analysis and bioinformatics analysis, after a rational design and prediction of potential enzymatic centers, base editors could be used to generate novel proteins with higher activity. Uniting ABE and CBE with a sgRNA fulling covering the protein coding sequence could allow direct evolution of protein (Li et al. 2020). Although not all types of nucleotide conversion are available, due to the lack of base editors for thymine and guanine, the editing window brings other possibilities for new protein mining (Li et al. 2020).

PE works for all types of nucleotides, and it was reported to induce nucleotide conversions, insertions and deletions (Anzalone et al. 2019). Moreover, PE could induce SVs like genomic deletions and insertions (Li et al. 2023a; Sun et al. 2024; Tao et al. 2022). The precision of PE tools make protein evolution more specific, and evolution through PE could focus on single amino acid scanning using a pegRNA pool to try all possibilities at a specific location (Xu et al. 2021a). Efficient PE for plants still under development, but it represents an exciting direction for crop improvement (Jiang et al. 2022, 2020).

### Developing plant regulators

Recent studies suggest that applying chemical treatments during high-temperature periods can substantially mitigate the damage caused by HS in plants. For instance, in rice, the exogenous application of spermidine not only improves the antioxidant system but also helps maintain high levels of endogenous spermidine and spermine, ensuring rice production under HS (Zhou et al. 2020). Similarly, applying 6-benzylaminopurine (6-BA), a synthetic cytokinin (CTK), alleviates heat-induced damage to spikelet fertility, spikelet number, and kernel weight (Wu et al. 2016). The application of brassinosteroids (BR), salicylic acid (SA), and ethylene precursors in rice plants has led to improvement in heat-triggered oxidative damage and morphological symptoms, highlighting the significant roles of these phytohormones in HS response (Chandrakala et al. 2013; Feng et al. 2018; Wu and Yang 2019). Phospholipids, which are fundamental constituents of cellular membranes, play crucial roles in plant temperature adaptation (Harwood 1991, 1994). Under HS, the unsaturation level of phospholipids in peanuts significantly decreases, while the levels of saturated phospholipids and monounsaturated phospholipids accumulated in the endoplasmic reticulum and plasma membrane of terrestrial plants increase (Higashi and Saito 2019; Zoong Lwe et al. 2020). Recent studies have also shown a direct correlation between phosphatidylcholine and phosphatidylethanolamine levels and temperature, indicating their potential role in maintaining membrane fluidity (Cheng et al. 2023; Qian et al. 2023). The efficacy of exogenous phospholipid application in alleviating high-temperature damage remains uncertain. Nevertheless, these studies indicate that the mechanisms governing crop responses to high temperatures cannot be fully understood in the short term, and the mass production of heat-tolerant varieties remains challenging. Developing low-cost and environmentally friendly plant regulators is another way that could significantly alleviate the impacts of HS.

## Conclusions and perspectives

Given the increasing challenges posed by climate change, studying crop tolerance to high temperatures is of utmost importance. While traditional breeding methods are valuable, they have limitations in addressing the complexity of heat tolerance. Past and present research has provided valuable insight into the genetic and physiological mechanisms of heat tolerance response. However, our understanding of crop heat response mechanisms and available genetic resources are limited, especially regarding sensitive stages like tillering, reproductive growth, and grain filling. The rapid development of genomics and gene editing technologies enables the rapid screening of major genes and the creation of new variations, offering possibilities for addressing heat tolerance effectively.

Global warming has led to rising nighttime temperatures, with high nighttime temperature also negatively affecting crop yield and grain quality (Xu et al. 2020b). In rice, high nighttime temperature reduces pollen viability, increases spikelet sterility, and induces membrane damage, resulting in significant yield losses (Cheng et al. 2009; Coast et al. 2015; Mohammed and Tarpley 2010; Xu et al. 2020b). Elevated nighttime temperatures disrupt gene expression timing in field conditions (Desai et al. 2021). Recent study identified glycine-rich RNA-binding proteins OsGRP3 and OsGRP162, as critical regulators of nighttime thermotolerance in rice (Yang et al. 2024). Acting downstream of the evening complex, OsGRP3 and OsGRP162 mediate diurnal thermotolerance by binding diverse mRNAs and interacting with spliceosomal components to regulate alternative splicing, ensuring yield stability under nighttime HS (Yang et al. 2024). Despite these progresses, key regulators driving diurnal thermotolerance under extreme high temperatures remain unidentified. Although genetic regions associated with high daytime temperature tolerance have been identified, efforts to uncover QTLs and causal genes linked to high nighttime temperature or combined high daytime and nighttime temperature stress are limited. Systematic approaches to pyramid these traits into breeding programs remain unexplored.

## Supplementary Information

Below is the link to the electronic supplementary material.

Supplementary file1 (DOCX 196 KB)

## Data Availability

Data sharing is not applicable to this article as no datasets were generated or analyzed as part of this study.
