# Supplementary material for: Emerging strategies to improve heat stress tolerance in crops
Source: aBIOTECH. 2025 Jan 24;6(1):97–115. doi: 10.1007/s42994-024-00195-z (PMC11889333; doi:10.1007/s42994-024-00195-z)
Supplement: Supplementary file 1 — Supplementary file1 (DOCX 196 KB) [file 42994_2024_195_MOESM1_ESM.docx]

**Supplemental Table S1 List of reported genes regulating crop heat stress response**

| Functioning stages | Simulation temperature | Genes/Proteins | Functions in HS | Favorable alleles | References |
| --- | --- | --- | --- | --- | --- |
| Rice | | | | | |
| V/R/F | 45°C;  38/35°C, day/night | *TT1* (*LOC_Os03g26970*);  The α2 subunit of the 26S proteasome. | Heat-induced; Protecting cells from HS through elimination of cytotoxic denatured proteins and maintenance of heat-response processes. | *TT1^CG14^*, from the *O. glaberrima.* | (Li et al. 2015) |
| V/R/F | 45°C | *TTL1* (*LOC_Os01g66970*);  A C3HC4 type domain containing transcription factor. | Heat-induced; Negatively regulating thermotolerance and grain size. Loss‐of‐function mutations in TTL1 enhances heat tolerance, and causes an increase in grain size by coordinating cell expansion and proliferation | *TTL1^hapL^*, mainly distributed in the *indica* subpopulation of *O. sativa.* | (Lin et al. 2023b) |
| V/R | 42°C | *TT2* (*Os03g0407400*);  A heterotrimeric G-protein γ subunit. | Inhibiting wax synthesis under HS through SCT1. | *TT2^HPS32^*, from the *japonica* subpopulation of *O. sativa*. | (Kan et al. 2022) |
| V/R | 42°C | *SCT1* (*LOC_Os03g09100*);  A Ca^2+^ sensing transcription factor. | Inhibiting wax synthesis under HS. |  | (Kan et al. 2022) |
| V/R | 28°C and 32°C | *WLP2* (*LOC_Os01g63220*);  A plastid-encoded RNA polymerase associated protein. | Protecting chloroplast development from HS. |  | (Lv et al. 2017) |
| V/R | 45 to 55°C;  44.9 /34.8°C, day/night | *OsHTG3* (*LOC_Os03g06630*);  A heat shock transcription factor. | Heat-induced; Encoding three splice variant proteins. A transcriptionally active form is alternatively spliced under HS. HTG3 regulates OsJAZ9, OsJAZ12 and other heat response genes to increase plant heat resistance. | *HTG3^M-^*, mainly distributed in the *indica* subpopulation of *O. sativa*. | (Cheng et al. 2015; Wu et al. 2022) |
| V/R | 45°C;  40/31°C, day/night | *SLG1* (*LOC_Os12g39840*);  The cytosolic tRNA 2-thiolation protein 2. | Heat-induced; SLG1-mediated thermotolerance is positively correlated with thiolated tRNA levels. | *SLG1^Ind^*, mainly distributed in the *indica* subpopulation of *O. sativa.* | (Xu et al. 2020b) |
| V/R | 42°C;  38/34°C, day/night. | *TT3.1* (*LOC_Os03g49900*);  A RING finger ubiquitin E3 ligase.  *TT3.2* (*LOC_Os03 g49940*);  A chloroplast precursor protein. | TT3.1 translocates during HS, leading to the ubiquitination of TT3.2 for vacuolar degradation. This process protects thylakoids from HS. | *TT3^CG14^*, from the *O. glaberrima.* | (Zhang et al. 2022a) |
| V/R | 44°C;  40/35°C, day/night | *OsEDS1* (*LOC_Os09g22450*);  A conserved triacylglycerol-like protein. | Heat-induced; Increasing thermotolerance by interacting with and promoting catalase-mediated H2O2 scavenging activities to control ROS homeostasis. |  | (Liao et al. 2023) |
| V/R | 43°C and 45°C | *ONAC023* (LOC_Os02g12310);  A NAC transcription factor. | Heat-induced; ONAC023 enhances drought and heat tolerance by translocating to the nucleus under stress, inducing genes involved in water transport, ROS homeostasis, and alternative splicing. |  | (Chang et al. 2024) |
| V/R | 45°C | *OsGRP3* (*LOC_Os03g46770*), *OsGRP162* (*LOC_Os12g43600*);  Glycine-rich RNA binding proteins. | Heat-induced; Regulating diurnal thermotolerance by binding to various mRNAs and interact with spliceosomal components to regulate alternative splicing. |  | (Yang et al. 2024) |
| V/R | 42°C | *OsGSA1* (*LOC_Os03g55040*);  A UDP-glucosyltransferase. | A positive grain size regulator that facilitates the accumulation of flavonoid glycosides and anthocyanins to eliminate the damage from HS. | *GSA1^WYJ^,* from the *japonica* subpopulation of *O. sativa*. | (Dong et al. 2020) |
| V | 38°C | *OsNCED3* (*LOC_Os03g44380*);  The 9-cis-epoxycarotenoid dioxygenase 1, a key enzyme in ABA biosynthesis. | Heat-induced; Mediating the heat-induced ABA accumulation in the seeds. |  | (Liu et al. 2019a) |
| V | 35°C | *OsNAA15* (LOC_Os01g43030);  A N-terminal acetyltransferase A. | Heat-induced; Reducing ROS production by regulating the N-terminal acetylation of glycolate oxidases, aiding rice growth in high temperatures. |  | (Li et al. 2024a) |
| V | 45°C | *OsMDHAR4*;  A monodehydroascorbate reductase. | Heat-induced; Promoting stomatal opening by reducing H_2_O_2_ content under HS. |  | (Liu et al. 2018) |
| V | 45°C | *OsHTAS* (*LOC_Os09g15430*);  A RING finger ubiquitin E3 ligase. | Heat-induced; Promoting H2O2-induced stomatal closure. |  | (Liu et al. 2015) |
| V | 42°C | *DST* (*LOC_Os03g57240*);  A Cys-2/His-2-type zinc finger protein. | Heat-induced; Promoting H2O2-induced stomatal closure. |  | (Cui et al. 2015; Ding et al. 2023) |
| V | 40°C | *RCA* (*LOC_Os11g47970*);  A rubisco activase. | RCA_L_ is heat-induced; Promoting photosynthetic acclimation to moderate HS. |  | (Qu et al. 2021) |
| V | 32°C | *OsFLN2* (*LOC_Os03g40550*);  A plastid-encoded RNA polymerase associated protein. | Protecting chloroplast development from HS. |  | (Qiu et al. 2018) |
| V | 42°C | *OsUBP21* (*LOC_Os11g36470*);  A ubiquitin-specific protease. | Heat-induced; Negatively regulating HS tolerance via deubiquitinating a set of proteins. |  | (Zhou et al. 2019) |
| V | 45°C | *WRKY10* (*LOC_Os01g09100*);  A WRKY transcription factor.  *VQ8* (*LOC_Os02g33600*);  A VQ motif containing protein. | Heat-induced; WRKY10 negatively regulates thermotolerance via ROS balance and hypersensitive response, while VQ8 has an antagonistic, positive role in thermotolerance. |  | (Chen et al. 2022) |
| V | 45°C | *OsbZIP74* (*LOC_Os06g41770*)  A bZIP transcription factor. | Heat triggers the unconventional splicing of bZIP74 mRNA, leading to the nuclear OsbZIP74 protein, an essential ER stress regulator influenced by heat and salicylic acid. |  | (Lu et al. 2012) |
| V | 45°C | *OsNTL3* (*LOC_Os01g15640*);  A membrane‐associated NAC transcription factor. | Heat-induced; OsbZIP74 up-regulates *OsNTL3* in response to heat and ER stresses. OsNTL3 relocates to the nucleus and regulates the expression of *OsbZIP74* and other genes involved in UPR under HS conditions. |  | (Liu et al. 2019c) |
| V | 45°C | *OsDOF27* (*LOC_Os10g35300*);  A Dof transcription factor. | Heat-induced; Overexpressing *OsDOF27* enhances rice seedling survival and seed germination rates under HS. |  | (Gandass et al. 2022) |
| V | 50°C | *OsANN1* (*LOC_Os02g51750*);  A calcium-binding protein, a member of the annexin protein family in rice. | Heat-induced; OsANN1 confers abiotic stress tolerance by modulating antioxidant accumulation under abiotic stress. |  | (Qiao et al. 2015) |
| V | 32°C | *TCM5* (*LOC_Os05g34460*);  A deg protease protein. | Heat-induced; TCM5 plays a crucial role in maintaining chloroplast development and PSII function under high temperatures. |  | (Zheng et al. 2016) |
| V | 45°C in rice;  38°C and 45°C in Arabidopsis. | *OsHIRP1* (*LOC_Os03g19020*);  A RING finger ubiquitin E3 ligase. | Heat-induced; OsHIRP1 ubiquitinates OsAKR4 and OsHRK1 under high temperature, and overexpressing it in Arabidopsis enhances plant thermotolerance. |  | (Kim et al. 2019) |
| V | 36°C | *OsNSUN2* (*LOC_Os09g29630*);  An RNA 5-methylcytosine methyltransferase. | Heat-induced; OsNSUN2 modulates m5C modification in mRNAs related to photosynthesis and detoxification, enhancing protein synthesis and thermotolerance. |  | (Tang et al. 2020) |
| V | 50°C | *OsERF115* (*LOC_Os08g41030*);  An ERF transcription factor. | Heat-induced; Overexpressing *OsERF115/AP2EREBP110* improves the thermotolerance of both seeds and vegetative growth plants. |  | (Park et al. 2021) |
| V | 40/30°C, day/night. | *OsGER4* (*LOC_Os01g18170*);  Germin-like protein. | Heat-induced; Maintaining root development under HS. |  | (Nguyen et al. 2023) |
| V | 42°C | *SNAC3* (*LOC_Os01g09550*);  A NAC transcription factor. | Heat-induced; Modulating ROS homeostasis via upregulating numerous ROS-scavenging genes. |  | (Fang et al. 2015) |
| V | 42°C and 47°C | *HSP101* (*LOC_Os05g44340*);  A heat shock protein.  *HSA32* (*LOC_Os06g46900*);  A HS-associated 32-kD protein. | Heat-induced; Prolonging the effect of heat acclimation in rice seedlings. The interplay between HSP101 and HSA32 also affects basal thermotolerance of rice seeds. |  | (Lin et al. 2014) |
| V | 42°C | *OsCNGC14* (*LOC_Os03g55100)*,  *OsCNGC16* (*LOC_Os05g42250*);  Cyclic nucleotide-gated ion channel proteins. | Heat-induced; Involved in the regulation of temperature stress-induced cytoplasmic calcium influx. |  | (Cui et al. 2020) |
| V | 35°C | *HES1* (*LOC_Os08g10600*);  A UDP-N-acetylglucosamine pyrophosphorylase. | Heat-induced; Enhancing heat tolerance via maintaining chloroplast function. |  | (Xia et al. 2022) |
| V | 42°C | *OsSGS3a* (*LOC_Os12g09580)*,  *OsSGS3b* (*LOC_Os12g09590*);  dsRNA-binding proteins. | Heat-repressed; OsSGS3a interacts with its homolog OsSGS3b and modulates the biogenesis of tasiRNA targeting ARFs. OsSGS3a/b positively, while OsARF3a/b and OsARF3la/lb negatively modulate thermotolerance. |  | (Gu et al. 2023) |
| V | 42°C | *OsHsp70CP1* (*LOC_Os05g23740*);  A chloroplast-localized heat shock protein. | Heat-induced; Maintaining chloroplast development under high temperatures. |  | (Kim and An 2013) |
| V | 32°C | *LS1* (*LOC_Os11g05570*);  The subunit of the RNase H2 complex. | LS1 is involved in DNA damage repair and plays an important role in maintaining genome stability and ROS homeostasis. |  | (Qiu et al. 2019) |
| V | 42°C | *SRL10* (*LOC_Os10g33970*);  A double-stranded RNA-binding protein. | Heat-induced; Enhancing thermotolerance by interacting with catalase isozyme B (CATB) and augmenting its H2O2 scavenging capacity. | *SRL10^hap3^*, mainly distributed in the *indica* and *Aus* subpopulations of *O. sativa*. | (Wang et al. 2023b) |
| V | 45°C | *ProDH* (*LOC_Os10g40360*);  A proline dehydrogenase. | Heat-repressed; Negatively regulating thermotolerance by modulating proline metabolism and ROS scavenging. |  | (Guo et al. 2020a) |
| V | 38°C and 45°C | *OsHCI1* (*LOC_Os10g30850*);  A RING E3 ligase. | Heat-induced; Facilitating nuclear-cytoplasmic trafficking of nuclear substrate proteins through monoubiquitination and acting as an inactivation mechanism for nuclear proteins during HS. |  | (Lim et al. 2013) |
| V | 45°C | *TOGR1* (*LOC_Os03g46610*);  A DEAD-box RNA helicase. | Heat-induced; Maintaining rRNA homeostasis under high temperature. |  | (Wang et al. 2016) |
| V | 45°C | *RGA1* (*LOC_Os05g26890*);  A heterotrimeric G protein α subunit. | Heat-repressed; Negatively regulating the thermotolerance in rice seedling plants through affecting carbohydrate and energy metabolism. |  | (Feng et al. 2023) |
| V | 45°C | *HTS1* (*LOC_Os04g30760*);  A thylakoid membrane-localized β-ketoacyl carrier protein reductase. | Heat-induced; Increasing thermotolerance by regulating fatty acid biosynthesis and stress signal transduction. |  | (Chen et al. 2021) |
| V | 45°C | *OsHBP1b* (*LOC_Os01g17260*);  A bZIP transcription factor. | Heat-induced; Enhancing chlorophyll content, boosting antioxidant capacity, and modulating stress response gene expression. |  | (Das et al. 2019) |
| V | 22 to 35°C | *AET1* (*LOC_Os05g45890*);  A tRNA^His^ guanylyltransferase. | Activating tRNA^His^ aminoacylation. AET1 interacts with RACK1A and eIF3h, regulating the translation of *OsARFs*. |  | (Chen et al. 2019) |
| V | 45°C | *PSL50* (*LOC_Os01g50770*);  A clathrin-associated adaptor protein complex 1. | Maintaining ROS homeostasis and photosynthetic system stability under HS. |  | (He et al. 2021) |
| V | 45°C in rice;  38/32°C, day/night in Arabidopsis | *OsDHSRP1* (*LOC_Os02g05692*);  A RING finger E3 ligase. | Heat-induced; Negatively regulating plant abiotic stress tolerance through the UPS, with OsGLYI-11.2 and OsACP1 as substrates of OsDHSRP1. |  | (Kim et al. 2020) |
| V | 37°C and 45°C in Arabidopsis | *OsCam1-1* (*LOC_Os03g20370*);  A salt-stress-responsive calmodulin. | Heat-induced; Mediating HS-triggered Ca^2+^ signaling transduced. |  | (Wu et al. 2012) |
| V | 38 °C | *PWL1* (*LOC_Os03g62180*);  A G-type lectin receptor-like kinase. | Enhancing thermotolerance possibly via modulating the ROS balance and decreasing heat-induced chloroplasts degradation. |  | (Xu et al. 2023) |
| R/F | 35/28°C, day/night. | *OsMADS7* (*LOC_Os08g41950*);  A MADS box protein. | Heat-induced; Constitutive suppression of *OsMADS7* stabilizes amylose content under high temperature stress but results in low spikelet fertility, while specific suppression of *OsMADS7* in endosperm could avoid it. |  | (Zhang et al. 2018) |
| R/F | 32°C;  32/32°C and 32/25°C, day/night | *OsNRT2.3* (*LOC_Os01g50820*);  A high-affinity nitrate transporter. | Heat-induced; Maintaining high yield and high nitrogen use efficiency under high temperatures. | *OsNRT2.3^HTNE-2^*, mainly distributed in the *Aus* subpopulation of *O. sativa*. | (Zhang et al. 2022b) |
| R/F | 30/23°C, 30/28°C, day/night | *MSD1* (*LOC_Os05g25850*);  A golgi/plastid-type manganese-superoxide dismutase. | Heat-induced; *MSD1* overexpression enhances ROS scavenging, chaperone activity, and quality control systems in rice grains during HS. |  | (Shiraya et al. 2015) |
| R | 34°C and 42°C | *OsFIE1* (*LOC_Os08g04290*);  An Esc-like core component of the polycomb repressive complex 2. | Increased under 34°C, repressed under 42°C; Regulating seed size under HS by controlling early endosperm development. |  | (Folsom et al. 2014) |
| R | 34°C | *HSP60-3B* (*LOC_Os10g32550*);  A Heat shock protein. | Heat-induced; Protecting pollen development by regulating starch accumulation and ROS levels at high temperatures. |  | (Lin et al. 2023a) |
| R | 38°C | *HTH5* (*LOC_Os05g05740*);  A pyridoxal phosphate homeostasis protein. | Heat-induced; Reducing ROS accumulation by increasing the pyridoxal 5'-phosphate (PLP) content, thereby enhancing the seed-setting rate of rice plants during HS. | *HTH5^HTT3^*, from the *O. rufipogon.* | (Cao et al. 2022) |
| R | 34°C | *OsMADS8* (*LOC_Os09g32948*);  A MADS box protein. | Maintaining pistil number and ovule initiation by modulating the expression of downstream genes in flower development pathways in a temperature-dependent manner. |  | (Shen et al. 2023) |
| R | 30°C and 34°C | *TAP* (*LOC_Os02g18370*);  A transposase-derived FAR1-RELATED SEQUENCE (FRS) protein. | Heat-induced; TAP independently and in conjunction with OsYAB regulates panicle and spikelet development under high temperature. |  | (Zhang et al. 2023) |
| R | 24/20°C, 29°/25°C, 33/29°C, 34/30°C, 37/33°C, and 37/37°C, day/night | *TSD1* (*LOC_Os02g18370*);  A poaceae-specific FHY3/FAR1 family transcription factor. | Heat-induced; Maintaining the normal initiation and development of spikelets at high temperatures via activating *YABBY* genes and interacting with them. |  | (Cai et al. 2023) |
| R | 42°C and 45°C | *OsHSBP1* (*LOC_Os09g20830*),  *OsHSBP2* (*LOC_Os06g16270*);  HSF binding proteins. | Heat-induced; Negatively regulating seed development under HS. |  | (Rana et al. 2012) |
| R | 35/30°C, and 40/30°C, day/night | *EG1* (*LOC_Os01g67430*);  A PLA1-type phospholipase. | Heat-induced; Promoting floral robustness against temperature fluctuation by safeguarding the expression of floral identify genes through a high temperature-dependent mitochondrial lipid pathway. |  | (Zhang et al. 2016) |
| R | 37/30°C, day/night | *OsNCED1* (*LOC_Os02g47510*);  A 9-cis-epoxycarotenoid dioxygenase. | Heat-induced; Overexpressing *OsNCED1* can improve the thermotolerance of rice at the heading and flowering stage by enhancing the antioxidant capacity. |  | (Zhou et al. 2022) |
| R | 36/26°C, day/night | *HYR* (*LOC_Os03g02650*);  An ethylene response factor. | Determining a morpho-physiological programme leading to higher grain yield under normal, drought and high-temperature stress conditions. |  | (Ambavaram et al. 2014) |
| R | 40 /35 °C, day/night | *OsIF* (*LOC_Os01g18840*);  An intermediate filament like protein. | Heat-induced; Overexpressing *OsIF* provides salinity and HS tolerance at the reproductive stage. |  | (Soda et al. 2018) |
| R | 35/30°C and 39/35°C, day/night | *OsMADS87* (*LOC_Os03g38610*);  A MADS-box family gene. | Heat-induced; Positively regulating seed size but negatively modulates thermotolerance. |  | (Chen et al. 2016) |
| F | 35°C | *ONAC127* (*LOC_Os11g31340*);  *ONAC129* (*LOC_Os11g31380*);  Seed-specific NAC domain transcription factors. | Heat-induced; Regulating grain filling by affecting sugar transportation and abiotic stress responses. |  | (Ren et al. 2021) |
| F | 35°C | *OsDML4* (*LOC_Os06g13070*);  A demethylase. | Increasing HS-induced chalkiness via the demethylation of *RISBZ1* and *RPBF*. |  | (Yan et al. 2022) |
| F | 35 /28°C, day/night. | *OsbZIP58* (*LOC_Os07g08420*);  A bZIP transcription factor. | Heat-induced; Enhancing transcription of seed storage protein genes and starch synthesis genes while reducing expression of starch hydrolyzing α-amylase genes under HS. |  | (Xu et al. 2020a) |
| F | 34/26°C, day/night | *SUS3* (*LOC_Os07g42490*);  A sucrose synthase. | Heat-induced; The upregulation of *SUS3* during ripening enhances high temperature tolerance in rice. | *SUS3^haba^*, from the *japonica* subpopulation of *O. sativa*. | (Takehara et al. 2018) |
| F | 24 to 35°C | *DG1* (*LOC_Os03g12790*);  A MATE protein, ABA efflux transporter. | Heat-induced; Mediating long-distance ABA transport, which activates starch synthesis genes and promotes grain filling. Both the DG1-mediated long-distance ABA transport efficiency and grain-filling phenotypes are temperature sensitive. |  | (Qin et al. 2021) |
| F | 36/32°C, day/night | *OsCG5* (*LOC_Os05g40850*);  A grain-specific, expressed protein of unknown function. | Heat-induced; Regulating natural variation for grain chalkiness under HS. Higher OsCG5 transcript level negatively correlates with grain chalkiness under HS |  | (Chandran et al. 2022) |
| F | 42°C;  35/28°C, day/night | *FLO24* (*LOC_Os03g31300*);  A heat shock protein 101. | Heat-induced; FLO24 acts as an important regulator of endosperm development, which might function in maintaining the activities of enzymes involved in starch biosynthesis in rice. |  | (Wu et al. 2024) |
| Maize | | | | | |
| V/R | 42°C in maize;  45°C in Arabidopsis. | *ZmDREB2A* (GenBank: *AB218832*);  An AP2/EREBP transcription factor. | Heat-induced; Regulating the expression of genes encoding late embryogenesis abundant proteins and HSPs. |  | (Qin et al. 2007) |
| V | 42°C | *ZmCDPK7*;  A calcium-dependent protein kinase. | Heat-induced; Regulating the expression and phosphorylation levels of sHSP17.4 and RBOHB. |  | (Zhao et al. 2021) |
| V | 37°C and 42°C | *ZmbZIP60* (*Zm00001d046718*);  A bZIP transcription factor. | Heat-induced; Upregulating UPR genes and a set of HSP genes. |  | (Li et al. 2020) |
| V | 37°C | *ZmHUG1* (*Zm00001d045336*);  A heat-inducible holdase-type molecular chaperone localized in the ER. | Heat-induced; Interacting with ZmPRA1.C1 and alleviates its heat-induced aggregation. |  | (Xie et al. 2022) |
| V | 42°C in maize;  44°C in Arabidopsis. | *ZmHSF01* (GenBank: *MK888854*);  A heat shock transcription factor. | Heat-induced; Upregulating HSP genes. |  | (Zhang et al. 2020) |
| V | 42°C in maize;  45°C in Arabidopsis. | *ZmHSF05* (GenBank: *MH845619*);  Heat shock transcription factor. | Heat-induced; Upregulating HSP genes. |  | (Li et al. 2019) |
| V | 42°C in Arabidopsis;  45°C in rice. | *ZmHSF11* (*Zm00001d052738*);  A heat shock transcription factor. | Heat-induced; Decreasing plant HS tolerance by increasing ROS levels and decreasing proline content. |  | (Qin et al. 2022) |
| V | 42°C | *ZmRPP13-LK3* (*Zm00001d045512*);  A Putative disease resistance RPP13-like protein 3. | Heat-induced; Catalyzing ATP for the production of cAMP and may be involved in ABA-regulated heat resistance. |  | (Yang et al. 2021) |
| V | 45°C in Arabidopsis;  42°C in maize. | *ZmWRKY106* (*Zm00001d012746*);  A WRKY transcription factor. | Heat-induced; May regulating stress-related genes through the ABA-signaling pathway |  | (Wang et al. 2018) |
| V | 37°C and 42°C | *ZmMPK20* (*Zm00001d039141*);  A mitogen-activated protein kinase. | Decreasing stomatal apertures, water loss rate. ZmMPK20 prevents ZmRIN2 degradation by inhibiting ZmRIN2 self-ubiquitination. |  | (Cheng et al. 2023) |
| V | 45°C | *ZmHSF20* (*Zm00001d026094*);  A heat shock transcription factor. | Heat-induced; Negatively regulating HS by binding and repressing the *Cellulose synthase A2 (ZmCesA2)* and three class A *HSF* genes expression. |  | (Li et al. 2024b) |
| R | 32°C and 35°C | *Ms42/ZmHSP101* (*Zm00001d038806*);  A heat shock protein. | Heat-induced; Function in RAD51 loading, DSB repair, and subsequent meiosis. |  | (Li et al. 2022) |
| R | 35°C | *INVAN6* (*Zm00001d015094*);  A cytosolic invertase that predominantly exists in PMCs and specifically hydrolyses sucrose. | Heat-induced; Maintaining anther glucose homeostasis by regulating glucose metabolism and transport genes to alleviate stress and support normal meiosis in pollen mother cells. |  | (Huang et al. 2022) |
| R | 32/28°C, day/night | *ZmFTSH10* (*Zm00001d010522*);  A mitochondria-localized ATP-dependent metalloprotease. | Maintaining reproductive meristem redox status and auxin homeostasis. |  | (Liu et al. 2019b) |
| Wheat | | | | | |
| V/R/F | 35°C in Arabidopsis;  37°C in wheat. | *sHsp26* (GenBank: *AF097657*);  A nuclear-encoded chloroplast small heat shock protein. | Heat-induced; Protecting PSII under HS conditions. |  | (Chauhan et al. 2012) |
| V/R | 42°C | *TaRca1* (GenBank: *KC776912*);  A rubisco activase. | Heat-induced; Regulating carbon assimilatory pathway under the HS. |  | (Kumar et al. 2016) |
| V/F | 40°C | *TaHAG1* (*TraesCS1D02G134200*);  A histone acetyltransferase. | Heat-induced; Interacting with TaNACL to regulate the transcription of *TaG1* and *TaPSBR1*. |  | (Lin et al. 2022) |
| V/F | 35°C and 42°C | *TaHSFA1-A* (*TraesCS4A02G322300*),  *TaHSFA1-B* (*TraesCS5B02G556200*),  *TaHSFA1-D* (*TraesCS5D02G553300)*;  Heat shock transcription factors. | Heat-induced; The SUMOylation of TaHSFA1 increases its transcriptional activation activity, but is suppressed by sustained heat exposure. TaHSFA1 may function by a thermosensitive interaction with TaHAG1. |  | (Wang et al. 2023a) |
| V | 36°C in wheat;  40°C in Arabidopsis. | *TaHSFA6f* (GenBank: *KJ774108*);  A heat shock transcription factor. | Heat-induced; Upregulating *HSP*s genes and a number of other HS protection genes. |  | (Bi et al. 2020; Xue et al. 2014) |
| V | 42°C in wheat;  45°C in Arabidopsis. | *TaWRKY1* (GenBank: *KT285206*),  *TaWRKY33* (GenBank: *KT285207*);  WRKY transcription factors. | Heat-induced; Upregulating stress-related genes. |  | (He et al. 2016) |
| V | 42°C | *TaHSFA6e;*  A heat shock transcription factor. | Heat-induced; Alternatively spliced under heat stress, HSFA6e-III increases the transcriptional activity of three *TaHSP70* genes more than TaHSFA6e-II. |  | (Wen et al. 2023) |
| V | 45°C in Arabidopsis | *TaBI-1.1* (*TraesCS6D01G077000*);  Bax inhibitor-1, a cell death suppressor. | Heat-induced; Regulating heat-responsive gene expression. |  | (Lu et al. 2018) |
| V | 45°C | *TaFER-5B* (GenBank: *KX025176*);  A ferritin. | Heat-induced; Protects cells from excessive ROS damage under HS |  | (Zang et al. 2017) |
| V | 40°C in wheat;  45°C in Arabidopsis. | *TaHSFA2e-5D* (GenBank: *OM735737*);  A heat shock transcription factor. | Heat-induced; Upregulating *HSPs* and other stress-related genes. |  | (Bi et al. 2022) |
| V | 37°C in wheat;  45°C in Arabidopsis. | *TaHSFA2-1*;  A heat shock transcription factor. | Heat-induced; Upregulating *HSPs* genes |  | (Liu et al. 2020) |
| V | 37°C in wheat;  45°C in Arabidopsis. | *TaHSFA2-10* (GenBank: *MK922287*);  A heat shock transcription factor. | Heat-induced; Upregulating *HSPs* genes |  | (Guo et al. 2020b) |
| V | 35°C | *TaHSFA6b* (*TraesCS4D02G276500*);  A heat shock transcription factor. | Heat-induced; Upregulating *HSPs* and other abiotic stress-responsive genes |  | (Poonia et al. 2020) |
| V | 45°C in Arabidopsis;  38°C and 42°C in wheat. | *TaMBF1c-7B* (GenBank: *GQ370008*);  A transcriptional coactivator that mediates transcriptional activation by physically bridging TFs. | Heat-induced; Colocalized with the stress granule complex and interacts with TaG3BP. TaMBF1c affects the translation efficiency of a subset of heat responsive genes. |  | (Tian et al. 2022) |
| V | 20°C | *TaCRK10* (GenBank: *MW076928*);  A cysteine-rich receptor-like kinase. | Heat- and *pst* inoculation-induced; Activating wheat high-temperature seedling-plant resistance to stripe rust through interacting with TaH2A.1 |  | (Wang et al. 2021) |
| V | 38°C | *Ta2CP* (*TraesCS2A02G297500*);  A 2-cysteine peroxiredoxin. | Heat-induced; Participating in chlorophyll metabolism by interacting with protochlorophyllide reductase b, TaPORB. |  | (Mishra et al. 2021) |
| V | 42°C | *TaSG-D1* (*TraesCS3A02G136500*);  A STKc_GSK3 kinase. | Heat-induced; Improving heat tolerance by enhancing phosphorylation and stability of downstream target TaPIF4. | TaSG-D1^E286K^, from the *Triticum sphaerococcum.* | (Cao et al. 2024) |
| V | 30°C in Arabidopsis;  42°C in rice;  30°C, 35°C, 40°C, 42°C, and 45°C in wheat. | *TaHSFA5* (*TraesCS6A02G098800*);  A heat shock transcription factor. | Heat-induced; Upregulating Hcxz  esponse genes. |  | (Samtani et al. 2023) |
| R/F | 37°C and 45°C in wheat;  45°C in Arabidopsis. | *TaHSFA2-7*;  A heat shock transcription factor. | *TaHSFA2-7-AS* is heat-induced; The splice variant *TaHSFA2-7-AS* is induced by high temperature, overexpression of *TaHSFA2-7-AS* in Arabidopsis results in enhanced tolerance to HS. |  | (Ma et al. 2023) |
| R | 38°C | *HSFA2h* (GenBank: *KP257297*);  A heat shock transcription factor. | Heat-induced; Possibly through upregulating *sHSP17* expression. |  | (Kumar et al. 2023) |
| F | 43°C and 45°C | *TaHSFC2a-B*;  A heat shock transcription factor. | Heat-induced; Upregulating heat protection genes. |  | (Hu et al. 2018) |

**Note:** “V” indicates the vegetative stage, “R” indicates the reproductive stage, and “F” indicates the grain filling stage.

**REFERENCES:**

Ambavaram MM et al. (2014) Coordinated regulation of photosynthesis in rice increases yield and tolerance to environmental stress. Nat Commun 5:5302. doi:<https://doi.org/10.1038/ncomms6302>

Bi H et al. (2022) Characterization of the wheat heat shock factor TaHsfA2e-5D conferring heat and drought tolerance in Arabidopsis. Int J Mol Sci 23:2784. doi:<https://doi.org/10.3390/ijms23052784>

Bi H, Zhao Y, Li H, Liu W (2020) Wheat heat shock factor TaHsfA6f increases ABA levels and enhances tolerance to multiple abiotic stresses in transgenic plants. Int J Mol Sci 21:3121. doi:<https://doi.org/10.3390/ijms21093121>

Cai Z et al. (2023) Thermo-Sensitive Spikelet Defects 1 acclimatizes rice spikelet initiation and development to high temperature. Plant Physiol 191:1684-1701. doi:<https://doi.org/10.1093/plphys/kiac576>

Cao J et al. (2024) Natural variation of STKc_GSK3 kinase TaSG-D1 contributes to heat stress tolerance in Indian dwarf wheat. Nat Commun 15:2097. doi:<https://doi.org/10.1038/s41467-024-46419-0>

Cao Z et al. (2022) Natural variation of HTH5 from wild rice, Oryza rufipogon Griff., is involved in conferring high-temperature tolerance at the heading stage. Plant Biotechnol J 20:1591-1605. doi:<https://doi.org/10.1111/pbi.13835>

Chandran AKN et al. (2022) Rice Chalky Grain 5 regulates natural variation for grain quality under heat stress. Front Plant Sci 13:1026472. doi:10.3389/fpls.2022.1026472

Chang Y et al. (2024) Stress-induced nuclear translocation of ONAC023 improves drought and heat tolerance through multiple processes in rice. Nat Commun 15:5877. doi:<https://doi.org/10.1038/s41467-024-50229-9>

Chauhan H, Khurana N, Nijhavan A, Khurana JP, Khurana P (2012) The wheat chloroplastic small heat shock protein (sHSP26) is involved in seed maturation and germination and imparts tolerance to heat stress. Plant Cell Environ 35:1912-1931. doi:<https://doi.org/10.1111/j.1365-3040.2012.02525.x>

Chen C, Begcy K, Liu K, Folsom JJ, Wang Z, Zhang C, Walia H (2016) Heat stress yields a unique MADS box transcription factor in determining seed size and thermal sensitivity. Plant Physiol 171:606-622. doi:<https://doi.org/10.1104/pp.15.01992>

Chen F et al. (2021) A β-ketoacyl carrier protein reductase confers heat tolerance via the regulation of fatty acid biosynthesis and stress signaling in rice. New Phytol 232:655-672. doi:<https://doi.org/10.1111/nph.17619>

Chen K et al. (2019) Translational regulation of plant response to high temperature by a dual-function tRNA(His) guanylyltransferase in Rice. Mol Plant 12:1123-1142. doi:<https://doi.org/10.1016/j.molp.2019.04.012>

Chen S, Cao H, Huang B, Zheng X, Liang K, Wang GL, Sun X (2022) The WRKY10‐VQ8 module safely and effectively regulates rice thermotolerance. Plant Cell Environ 45:2126-2144. doi:<https://doi.org/10.1111/pce.14329>

Cheng C et al. (2023) Maize MITOGEN-ACTIVATED PROTEIN KINASE 20 mediates high-temperature-regulated stomatal movement. Plant Physiol 193:2788-2805. doi:<https://doi.org/10.1093/plphys/kiad488>

Cheng Q et al. (2015) An alternatively spliced heat shock transcription factor, OsHSFA2dI, functions in the heat stress-induced unfolded protein response in rice. Plant Biol 17:419-429. doi:<https://doi.org/10.1111/plb.12267>

Cui LG, Shan JX, Shi M, Gao JP, Lin HX (2015) DCA1 Acts as a Transcriptional Co-activator of DST and Contributes to Drought and Salt Tolerance in Rice. PLoS Genet 11:e1005617. doi:<https://doi.org/10.1371/journal.pgen.1005617>

Cui Y et al. (2020) CYCLIC NUCLEOTIDE-GATED ION CHANNELs 14 and 16 Promote Tolerance to Heat and Chilling in Rice. Plant Physiol 183:1794-1808. doi:<https://doi.org/10.1104/pp.20.00591>

Das P, Lakra N, Nutan KK, Singla-Pareek SL, Pareek A (2019) A unique bZIP transcription factor imparting multiple stress tolerance in Rice. Rice 12:58. doi:<https://doi.org/10.1186/s12284-019-0316-8>

Ding Y et al. (2023) Rice DST transcription factor negatively regulates heat tolerance through ROS-mediated stomatal movement and heat-responsive gene expression. Front Plant Sci 14:1068296. doi:<https://doi.org/10.3389/fpls.2023.1068296>

Dong N-Q et al. (2020) UDP-glucosyltransferase regulates grain size and abiotic stress tolerance associated with metabolic flux redirection in rice. Nat Commun 11:2629. doi:<https://doi.org/10.1038/s41467-020-16403-5>

Fang Y, Liao K, Du H, Xu Y, Song H, Li X, Xiong L (2015) A stress-responsive NAC transcription factor SNAC3 confers heat and drought tolerance through modulation of reactive oxygen species in rice. J Exp Bot 66:6803-6817. doi:<https://doi.org/10.1093/jxb/erv386>

Feng B et al. (2023) RGA1 negatively regulates thermo-tolerance by affecting carbohydrate metabolism and the energy supply in Rice. Rice 16:32. doi:<https://doi.org/10.1186/s12284-023-00649-w>

Folsom JJ, Begcy K, Hao X, Wang D, Walia H (2014) Rice fertilization-Independent Endosperm1 regulates seed size under heat stress by controlling early endosperm development. Plant Physiol 165:238-248. doi:<https://doi.org/10.1104/pp.113.232413>

Gandass N, Kajal, Salvi P (2022) Intrinsically disordered protein, DNA binding with one finger transcription factor (OsDOF27) implicates thermotolerance in yeast and rice. Front Plant Sci 13:956299. doi:<https://doi.org/10.3389/fpls.2022.956299>

Gu X et al. (2023) The OsSGS3-tasiRNA-OsARF3 module orchestrates abiotic-biotic stress response trade-off in rice. Nat Commun 14:4441. doi:<https://doi.org/10.1038/s41467-023-40176-2>

Guo M, Zhang X, Liu J, Hou L, Liu H, Zhao X (2020a) OsProDH negatively regulates thermotolerance in rice by modulating proline metabolism and reactive oxygen species scavenging. Rice 13:61. doi:<https://doi.org/10.1186/s12284-020-00422-3>

Guo XL et al. (2020b) Heat-response patterns of the heat shock transcription factor family in advanced development stages of wheat (Triticum aestivum L.) and thermotolerance-regulation by TaHsfA2-10. BMC Plant Biol 20:364. doi:<https://doi.org/10.1186/s12870-020-02555-5>

He GH et al. (2016) Drought-responsive WRKY transcription factor genes TaWRKY1 and TaWRKY33 from wheat confer drought and/or heat resistance in Arabidopsis. BMC Plant Biol 16:116. doi:<https://doi.org/10.1186/s12870-016-0806-4>

He Y, Zhang X, Shi Y, Xu X, Li L, Wu JL (2021) PREMATURE SENESCENCE LEAF 50 promotes heat stress tolerance in rice (Oryza sativa L.). Rice 14:53. doi:<https://doi.org/10.1186/s12284-021-00493-w>

Hu XJ et al. (2018) Heat shock factor C2a serves as a proactive mechanism for heat protection in developing grains in wheat via an ABA-mediated regulatory pathway. Plant Cell Environ 41:79-98. doi:<https://doi.org/10.1111/pce.12957>

Huang W et al. (2022) Maize cytosolic invertase INVAN6 ensures faithful meiotic progression under heat stress. New Phytol 236:2172-2188. doi:<https://doi.org/10.1111/nph.18490>

Kan Y, Mu XR, Zhang H, Gao J, Shan JX, Ye WW, Lin HX (2022) TT2 controls rice thermotolerance through SCT1-dependent alteration of wax biosynthesis. Nat Plants 8:53-67. doi:<https://doi.org/10.1038/s41477-021-01039-0>

Kim JH, Lim SD, Jang CS (2019) Oryza sativa heat-induced RING finger protein 1 (OsHIRP1) positively regulates plant response to heat stress. Plant Mol Biol 99:545-559. doi:<https://doi.org/10.1007/s11103-019-00835-9>

Kim JH, Lim SD, Jang CS (2020) Oryza sativa drought-, heat-, and salt-induced RING finger protein 1 (OsDHSRP1) negatively regulates abiotic stress-responsive gene expression. Plant Mol Biol 103:235-252. doi:<https://doi.org/10.1007/s11103-020-00989-x>

Kim SR, An G (2013) Rice chloroplast-localized heat shock protein 70, OsHsp70CP1, is essential for chloroplast development under high-temperature conditions. J Plant Physiol 170:854-863. doi:<https://doi.org/10.1016/j.jplph.2013.01.006>

Kumar RR et al. (2023) Transcriptional regulation of Small Heat Shock Protein 17 (sHSP-17) by Triticum aestivum HSFA2h transcription factor confers tolerance in arabidopsis under heat stress. Plants-Basel 12:3598. doi:<https://doi.org/10.3390/plants12203598>.

Kumar RR et al. (2016) Identification of putative RuBisCo activase (TaRca1)-the catalytic chaperone regulating carbon assimilatory pathway in wheat (Triticum aestivum) under the heat stress. Front Plant Sci 7:986. doi:<https://doi.org/10.3389/fpls.2016.00986>

Li G-l et al. (2019) ZmHsf05, a new heat shock transcription factor from Zea mays L. improves thermotolerance in Arabidopsis thaliana and rescues thermotolerance defects of the athsfa2 mutant. Plant Sci 283:375-384. doi:<https://doi.org/10.1016/j.plantsci.2019.03.002>

Li X et al. (2024a) N-terminal acetylation orchestrates glycolate-mediated ROS homeostasis to promote rice thermoresponsive growth. New Phytol. doi:<https://doi.org/10.1111/nph.19928>

Li XM et al. (2015) Natural alleles of a proteasome α2 subunit gene contribute to thermotolerance and adaptation of African rice. Nat Genet 47:827-833. doi:<https://doi.org/10.1038/ng.3305>

Li Y et al. (2022) Heat shock protein 101 contributes to the thermotolerance of male meiosis in maize. Plant Cell 34:3702-3717. doi:<https://doi.org/10.1093/plcell/koac184>

Li Z et al. (2024b) The Heat shock factor 20–HSF4–Cellulose synthase A2 module regulates heat stress tolerance in maize. Plant Cell:koae106. doi:<https://doi.org/10.1093/plcell/koae106>

Li Z, Tang J, Srivastava R, Bassham DC, Howell SH (2020) The Transcription Factor bZIP60 Links the Unfolded Protein Response to the Heat Stress Response in Maize. Plant Cell 32:3559-3575. doi:<https://doi.org/10.1105/tpc.20.00260>

Liao M et al. (2023) ENHANCED DISEASE SUSCEPTIBILITY 1 promotes hydrogen peroxide scavenging to enhance rice thermotolerance. Plant Physiol 192:3106-3119. doi:<https://doi.org/10.1093/plphys/kiad257>

Lim SD, Cho HY, Park YC, Ham DJ, Lee JK, Jang CS (2013) The rice RING finger E3 ligase, OsHCI1, drives nuclear export of multiple substrate proteins and its heterogeneous overexpression enhances acquired thermotolerance. J Exp Bot 64:2899-2914. doi:<https://doi.org/10.1093/jxb/ert143>

Lin J et al. (2022) Histone acetyltransferase TaHAG1 interacts with TaNACL to promote heat stress tolerance in wheat. Plant Biotechnol J 20:1645-1647. doi:<https://doi.org/10.1111/pbi.13881>

Lin MY, Chai KH, Ko SS, Kuang LY, Lur HS, Charng YY (2014) A positive feedback loop between HEAT SHOCK PROTEIN101 and HEAT STRESS-ASSOCIATED 32-KD PROTEIN modulates long-term acquired thermotolerance illustrating diverse heat stress responses in rice varieties. Plant Physiol 164:2045-2053. doi:<https://doi.org/10.1104/pp.113.229609>

Lin S et al. (2023a) Rice HEAT SHOCK PROTEIN60-3B maintains male fertility under high temperature by starch granule biogenesis. Plant Physiol 192:2301-2317. doi:<https://doi.org/10.1093/plphys/kiad136>

Lin Y et al. (2023b) Identification of natural allelic variation in TTL1 controlling thermotolerance and grain size by a rice super pan-genome. J Integr Plant Biol. doi:<https://doi.org/10.1111/jipb.13568>

Liu J, Hasanuzzaman M, Wen H, Zhang J, Peng T, Sun H, Zhao Q (2019a) High temperature and drought stress cause abscisic acid and reactive oxygen species accumulation and suppress seed germination growth in rice. Protoplasma 256:1217-1227. doi:<https://doi.org/10.1007/s00709-019-01354-6>

Liu J et al. (2018) Suppression of OsMDHAR4 enhances heat tolerance by mediating H(2)O(2)-induced stomatal closure in rice plants. Rice 11:38. doi:<https://doi.org/10.1186/s12284-018-0230-5>

Liu J et al. (2015) The RING Finger Ubiquitin E3 Ligase OsHTAS Enhances Heat Tolerance by Promoting H2O2-Induced Stomatal Closure in Rice. Plant Physiol 170:429-443. doi:<https://doi.org/10.1104/pp.15.00879>

Liu Q et al. (2019b) NEEDLE1 encodes a mitochondria localized ATP-dependent metalloprotease required for thermotolerant maize growth. Proc Natl Acad Sci U S A 116:19736-19742. doi:<https://doi.org/10.1073/pnas.1907071116>

Liu XH, Lyu YS, Yang W, Yang ZT, Lu SJ, Liu JX (2019c) A membrane‐associated NAC transcription factor OsNTL3 is involved in thermotolerance in rice. Plant Biotechnol J 18:1317-1329. doi:<https://doi.org/10.1111/pbi.13297>

Liu Z et al. (2020) TaHsfA2-1, a new gene for thermotolerance in wheat seedlings: Characterization and functional roles. J Plant Physiol 246-247:153135. doi:<https://doi.org/10.1016/j.jplph.2020.153135>

Lu P-P et al. (2018) Wheat Bax Inhibitor-1 interacts with TaFKBP62 and mediates response to heat stress. BMC Plant Biol 18:259. doi:<https://doi.org/10.1186/s12870-018-1485-0>

Lu SJ, Yang ZT, Sun L, Sun L, Song ZT, Liu JX (2012) Conservation of IRE1-regulated bZIP74 mRNA unconventional splicing in rice (Oryza sativa L.) involved in ER stress responses. Mol Plant 5:504-514. doi:<https://doi.org/10.1093/mp/ssr115>

Lv Y et al. (2017) White Leaf and Panicle 2, encoding a PEP-associated protein, is required for chloroplast biogenesis under heat stress in rice. J Exp Bot 68:5147-5160. doi:<https://doi.org/10.1093/jxb/erx332>

Ma Z et al. (2023) Alternative Splicing of TaHsfA2-7 Is Involved in the Improvement of Thermotolerance in Wheat. Int J Mol Sci 24:1014. doi:<https://doi.org/10.3390/ijms24021014>

Mishra D, Shekhar S, Chakraborty S, Chakraborty N (2021) Wheat 2-Cys peroxiredoxin plays a dual role in chlorophyll biosynthesis and adaptation to high temperature. Plant J 105:1374-1389. doi:<https://doi.org/10.1111/tpj.15119>

Nguyen TT, Pham DT, Nguyen NH, Do PT, To HTM (2023) The Germin-like protein gene OsGER4 is involved in heat stress response in rice root development. Funct Integr Genomics 23:271. doi:<https://doi.org/10.1007/s10142-023-01201-1>

Park S-I et al. (2021) The OsERF115/AP2EREBP110 Transcription Factor Is Involved in the Multiple Stress Tolerance to Heat and Drought in Rice Plants. Int J Mol Sci 22:7181. doi:<https://doi.org/10.3390/ijms22137181>.

Poonia AK, Mishra SK, Sirohi P, Chaudhary R, Kanwar M, Germain H, Chauhan H (2020) Overexpression of wheat transcription factor (TaHsfA6b) provides thermotolerance in barley. Planta 252:53. doi:<https://doi.org/10.1007/s00425-020-03457-4>

Qiao B et al. (2015) A calcium-binding protein, rice annexin OsANN1, enhances heat stress tolerance by modulating the production of H2O2. J Exp Bot 66:5853-5866. doi:<https://doi.org/10.1093/jxb/erv294>

Qin F et al. (2007) Regulation and functional analysis of ZmDREB2A in response to drought and heat stresses in Zea mays L. Plant J 50:54-69. doi:<https://doi.org/10.1111/j.1365-313X.2007.03034.x>

Qin P et al. (2021) Leaf-derived ABA regulates rice seed development via a transporter-mediated and temperature-sensitive mechanism. Sci Adv 7:eabc8873. doi:<https://doi.org/10.1126/sciadv.abc8873>

Qin Q, Zhao Y, Zhang J, Chen L, Si W, Jiang H (2022) A maize heat shock factor ZmHsf11 negatively regulates heat stress tolerance in transgenic plants. BMC Plant Biol 22:406. doi:<https://doi.org/10.1186/s12870-022-03789-1>

Qiu Z et al. (2018) The newly identified heat-stress sensitive albino 1 gene affects chloroplast development in rice. Plant Sci 267:168-179. doi:<https://doi.org/10.1016/j.plantsci.2017.11.015>

Qiu Z et al. (2019) DNA damage and reactive oxygen species cause cell death in the rice local lesions 1 mutant under high light and high temperature. New Phytol 222:349-365. doi:<https://doi.org/10.1111/nph.15597>

Qu Y et al. (2021) Overexpression of both Rubisco and Rubisco activase rescues rice photosynthesis and biomass under heat stress. Plant Cell Environ 44:2308-2320. doi:<https://doi.org/10.1111/pce.14051>

Rana RM, Dong S, Tang H, Ahmad F, Zhang H (2012) Functional analysis of OsHSBP1 and OsHSBP2 revealed their involvement in the heat shock response in rice (Oryza sativa L.). J Exp Bot 63:6003-6016. doi:<https://doi.org/10.1093/jxb/ers245>

Ren Y et al. (2021) A heat stress responsive NAC transcription factor heterodimer plays key roles in rice grain filling. J Exp Bot 72:2947-2964. doi:<https://doi.org/10.1093/jxb/erab027>

Samtani H, Sharma A, Khurana P (2023) Ectopic overexpression of TaHsfA5 promotes thermomorphogenesis in Arabidopsis thaliana and thermotolerance in Oryza sativa. Plant Mol Biol 112:225-243. doi:<https://doi.org/10.1007/s11103-023-01355-3>

Shen C et al. (2023) MADS8 is indispensable for female reproductive development at high ambient temperatures in cereal crops. Plant Cell 36:65-84. doi:<https://doi.org/10.1093/plcell/koad246>

Shiraya T et al. (2015) Golgi/plastid-type manganese superoxide dismutase involved in heat-stress tolerance during grain filling of rice. Plant Biotechnol J 13:1251-1263. doi:<https://doi.org/10.1111/pbi.12314>

Soda N, Gupta BK, Anwar K, Sharan A, Govindjee, Singla-Pareek SL, Pareek A (2018) Rice intermediate filament, OsIF, stabilizes photosynthetic machinery and yield under salinity and heat stress. Sci Rep 8:4072. doi:<https://doi.org/10.1038/s41598-018-22131-0>

Takehara K et al. (2018) Thermo-responsive allele of sucrose synthase 3 (Sus3) provides high-temperature tolerance during the ripening stage in rice (Oryza sativa L.). Breed Sci 68:336-342. doi:<https://doi.org/10.1270/jsbbs.18007>

Tang Y et al. (2020) OsNSUN2-mediated 5-methylcytosine mRNA modification enhances rice adaptation to high temperature. Dev Cell 53:272-286. doi:<https://doi.org/10.1016/j.devcel.2020.03.009>

Tian X et al. (2022) Stress granule-associated TaMBF1c confers thermotolerance through regulating specific mRNA translation in wheat (Triticum aestivum). New Phytol 233:1719-1731. doi:<https://doi.org/10.1111/nph.17865>

Wang CT et al. (2018) Maize WRKY transcription factor ZmWRKY106 confers drought and heat tolerance in transgenic plants. Int J Mol Sci 19:3046. doi:<https://doi.org/10.3390/ijms19103046>

Wang D, Qin B, Li X, Tang D, Zhang Y, Cheng Z, Xue Y (2016) Nucleolar DEAD-Box RNA Helicase TOGR1 Regulates Thermotolerant Growth as a Pre-rRNA Chaperone in Rice. PLoS Genet 12:e1005844. doi:<https://doi.org/10.1371/journal.pgen.1005844>

Wang H et al. (2023a) Thermosensitive SUMOylation of TaHsfA1 defines a dynamic ON/OFF molecular switch for the heat stress response in wheat. Plant Cell 35:3889-3910. doi:<https://doi.org/10.1093/plcell/koad192>

Wang J, Wang J, Li J, Shang H, Chen X, Hu X (2021) The RLK protein TaCRK10 activates wheat high-temperature seedling-plant resistance to stripe rust through interacting with TaH2A.1. Plant J 108:1241-1255. doi:<https://doi.org/10.1111/tpj.15513>

Wang J et al. (2023b) SEMI-ROLLED LEAF 10 stabilizes catalase isozyme B to regulate leaf morphology and thermotolerance in rice (Oryza sativa L.). Plant Biotechnol J 21:819-838. doi:<https://doi.org/10.1111/pbi.13999>

Wen J et al. (2023) Alternative splicing of TaHSFA6e modulates heat shock protein-mediated translational regulation in response to heat stress in wheat. New Phytol 239:2235-2247. doi:<https://doi.org/10.1111/nph.19100>

Wu H et al. (2024) FLOURY ENDOSPERM24, a heat shock protein 101 (HSP101), is required for starch biosynthesis and endosperm development in rice. New Phytol. doi:<https://doi.org/10.1111/nph.19761>

Wu HC, Luo DL, Vignols F, Jinn TL (2012) Heat shock-induced biphasic Ca(2+) signature and OsCaM1-1 nuclear localization mediate downstream signalling in acquisition of thermotolerance in rice (Oryza sativa L.). Plant Cell Environ 35:1543-1557. doi:<https://doi.org/10.1111/j.1365-3040.2012.02508.x>

Wu N et al. (2022) A MITE variation-associated heat-inducible isoform of a heat-shock factor confers heat tolerance through regulation of JASMONATE ZIM-DOMAIN genes in rice. New Phytol 234:1315-1331. doi:<https://doi.org/10.1111/nph.18068>

Xia S et al. (2022) UDP-N-acetylglucosamine pyrophosphorylase enhances rice survival at high temperature. New Phytol 233:344-359. doi:<https://doi.org/10.1111/nph.17768>

Xie C et al. (2022) Maize HEAT UP-REGULATED GENE 1 plays vital roles in heat stress tolerance. J Exp Bot 73:6417-6433. doi:<https://doi.org/10.1093/jxb/erac262>

Xu H et al. (2020a) High temperature inhibits the accumulation of storage materials by inducing alternative splicing of OsbZIP58 during filling stage in rice. Plant Cell Environ 43:1879-1896. doi:<https://doi.org/10.1111/pce.13779>

Xu J et al. (2023) PWL1, a G-type lectin receptor-like kinase, positively regulates leaf senescence and heat tolerance but negatively regulates resistance to Xanthomonas oryzae in rice. Plant Biotechnol J 21:2525-2545. doi:<https://doi.org/10.1111/pbi.14150>

Xu Y, Zhang L, Ou S, Wang R, Wang Y, Chu C, Yao S (2020b) Natural variations of SLG1 confer high-temperature tolerance in indica rice. Nat Commun 11:5441. doi:<https://doi.org/10.1038/s41467-020-19320-9>

Xue G-P, Drenth J, McIntyre CL (2014) TaHsfA6f is a transcriptional activator that regulates a suite of heat stress protection genes in wheat (Triticum aestivum L.) including previously unknown Hsf targets. J Exp Bot 66:1025-1039. doi:<https://doi.org/10.1093/jxb/eru462>

Yan Y et al. (2022) A new demethylase gene, OsDML4, is involved in high temperature-increased grain chalkiness in rice. J Exp Bot 73:7273-7284. doi:<https://doi.org/10.1093/jxb/erac367>

Yang C, Luo A, Lu HP, Davis SJ, Liu JX (2024) Diurnal regulation of alternative splicing associated with thermotolerance in rice by two glycine-rich RNA-binding proteins. Sci Bull 69:59-71. doi:<https://doi.org/10.1016/j.scib.2023.11.046>

Yang H et al. (2021) A new adenylyl cyclase, putative disease-resistance RPP13-like protein 3, participates in abscisic acid-mediated resistance to heat stress in maize. J Exp Bot 72:283-301. doi:<https://doi.org/10.1093/jxb/eraa431>

Zang X et al. (2017) Overexpression of wheat ferritin gene TaFER-5B enhances tolerance to heat stress and other abiotic stresses associated with the ROS scavenging. BMC Plant Biol 17:14. doi:<https://doi.org/10.1186/s12870-016-0958-2>

Zhang B et al. (2016) A high temperature-dependent mitochondrial lipase EXTRA GLUME1 promotes floral phenotypic robustness against temperature fluctuation in rice (Oryza sativa L.). PLoS Genet 12:e1006152. doi:<https://doi.org/10.1371/journal.pgen.1006152>

Zhang H et al. (2020) Functional characterization of maize heat shock transcription factor gene ZmHsf01 in thermotolerance. PeerJ 8:e8926. doi:<https://doi.org/10.7717/peerj.8926>

Zhang H, Xu H, Feng M, Zhu Y (2018) Suppression of OsMADS7 in rice endosperm stabilizes amylose content under high temperature stress. Plant Biotechnol J 16:18-26. doi:<https://doi.org/10.1111/pbi.12745>

Zhang H et al. (2022a) A genetic module at one locus in rice protects chloroplasts to enhance thermotolerance. Science 376:1293-1300. doi:<https://doi.org/10.1126/science.abo5721>

Zhang P et al. (2023) THERMOSENSITIVE BARREN PANICLE (TAP) is required for rice panicle and spikelet development at high ambient temperature. New Phytol 237:855-869. doi:<https://doi.org/10.1111/nph.18551>

Zhang Y et al. (2022b) High-temperature adaptation of an OsNRT2.3 allele is thermoregulated by small RNAs. Sci Adv 8:eadc9785. doi:<https://doi.org/10.1126/sciadv.adc9785>

Zhao Y et al. (2021) The calcium-dependent protein kinase ZmCDPK7 functions in heat-stress tolerance in maize. J Integr Plant Biol 63:510-527. doi:<https://doi.org/10.1111/jipb.13056>

Zheng K et al. (2016) The Rice TCM5 Gene Encoding a Novel Deg Protease Protein is Essential for Chloroplast Development under High Temperatures. Rice 9. doi:<https://doi.org/10.1186/s12284-016-0086-5>

Zhou H et al. (2019) A Quantitative Proteomics Study of Early Heat‐Regulated Proteins by Two‐Dimensional Difference Gel Electrophoresis Identified OsUBP21 as a Negative Regulator of Heat Stress Responses in Rice. Proteomics 19:e1900153. doi:<https://doi.org/10.1002/pmic.201900153>

Zhou H et al. (2022) Comparative Analysis of Heat-Tolerant and Heat-Susceptible Rice Highlights the Role of OsNCED1 Gene in Heat Stress Tolerance. Plants-Basel 11. doi:<https://doi.org/10.3390/plants11081062>
